# Reef Cover, a coral reef classification for global habitat mapping from remote sensing

**DOI:** 10.1038/s41597-021-00958-z

**Published:** 2021-08-02

**Authors:** Emma V. Kennedy, Chris M. Roelfsema, Mitchell B. Lyons, Eva M. Kovacs, Rodney Borrego-Acevedo, Meredith Roe, Stuart R. Phinn, Kirk Larsen, Nicholas J. Murray, Doddy Yuwono, Jeremy Wolff, Paul Tudman

**Affiliations:** 1 Remote Sensing Research Centre, School of Earth and Environmental Sciences, University of Queensland, Brisbane, Australia; 2 Australian Institute of Marine Science, Townsville, Queensland Australia; 3 Vulcan Inc, Washington, 98104 USA; 4 College of Science and Engineering, James Cook University, Townsville, Queensland Australia

**Keywords:** Macroecology, Geomorphology, Marine biology, Environmental sciences, Coral reefs

## Abstract

Coral reef management and conservation stand to benefit from improved high-resolution global mapping. Yet classifications underpinning large-scale reef mapping to date are typically poorly defined, not shared or region-specific, limiting end-users’ ability to interpret outputs. Here we present *Reef Cover*, a coral reef geomorphic zone classification, developed to support both producers and end-users of global-scale coral reef habitat maps, in a transparent and version-based framework. Scalable classes were created by focusing on attributes that can be observed remotely, but whose membership rules also reflect deep knowledge of reef form and functioning. Bridging the divide between earth observation data and geo-ecological knowledge of reefs, *Reef Cover* maximises the trade-off between applicability at global scales, and relevance and accuracy at local scales. Two case studies demonstrate application of the *Reef Cover* classification scheme and its scientific and conservation benefits: 1) detailed mapping of the *Cairns Management Region* of the Great Barrier Reef to support management and 2) mapping of the Caroline and Mariana Island chains in the Pacific for conservation purposes.

## Abstract (summary)

**Table:** 

Measurement(s)	habitat
Technology Type(s)	satellite imaging • digital curation
Sample Characteristic - Organism	Anthozoa
Sample Characteristic - Environment	marine biome • coral reef
Sample Characteristic - Location	global

Machine-accessible metadata file describing the reported data: 10.6084/m9.figshare.14397182

## Background & Summary

Enhanced earth observation and analytical capabilities have revolutionised the way we view our planet, allowing a global perspective that has the potential to dramatically influence the way humanity manages finite planetary resources^1^. Yet for coral reefs, a valuable and rapidly degrading ecosystem^2,3^, these accelerating capabilities to collect and analyse data are not often translated into improved environmental outcomes^4^. One barrier is the transfer of insights gained from remote sensing data from producers (remote sensing scientists) to end-users (reef managers, policy makers, conservation practitioners). Translation of remotely collected observations of coral reefs into user-friendly spatial information for investigation, monitoring, planning and management requires a classification system to discretise continuous data about natural phenomena into spatial units of manageable information.

Classification is a key process for making data accessible to people that need it^5^. Maps are an efficient way to share large amounts of spatial data, but to be effective they must be underpinned by a classification framework that a) follows a clear and transparent rationale (*transparency*), b) defines classes that are meaningful for the application (*relevance*), c) is clearly and unambiguously described (*clarity*) and is d) discoverable and intuitive to interpret end-users (*accessibility*). The latter is particularly important if information is to be meaningfully integrated into practical solutions founded on map data^5^.

**Fig. 1:**
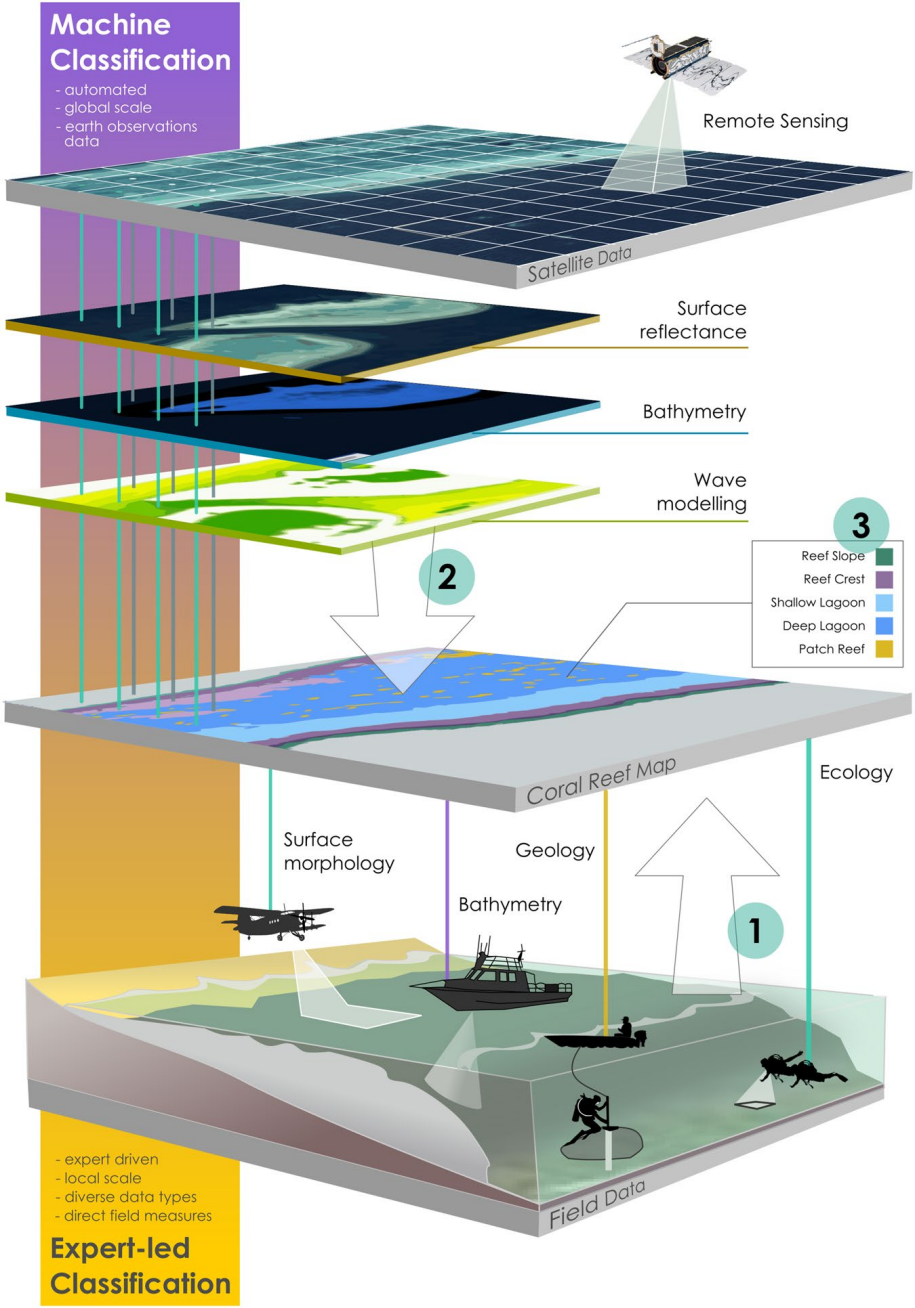
Different scales (global vs local) and scientific approaches (direct field measurements vs remote satellite observations) for capturing natural variability of morphological reef features can shape our understanding of how reefs are structured. Coral reef habitat maps, like the *Allen Coral Atlas*, NOAA’s *Biogeography of Coral Reef* maps, *Living Oceans Foundation* and *Millennium Coral Reef Mapping Project*, aim to distil the vibrant natural diversity of coral reef ecosystems into clustered information that is relevant, interpretable and useful to humans, so regional-to-global scale patterns of variability can be widely disseminated. A classification system for coral reef zones should try to integrate (1) decades of local scientific knowledge on coral reef systems, with the (2) global-scale information that is becoming more accessible from satellite sensors and information derived from these products, to (3) generate map classes that can inform understanding or management of coral reefs.

Classification of coral reef geomorphology has typically occurred using two broad approaches, which are largely congruent^6^, but diverge in places owing to the disciplinary approach, methodology employed, datasets used, and the scale and scope of investigation. For centuries, traditional reef classification has involved an *a priori* grouping of natural features into classes based on detailed field observations and expert knowledge, often drawing from multiple disciplines, and making use of diverse ecological and geological datasets (e.g. drill cores, bathymetry readings, ecological benthic data) and natural history theory (*Expert-led* classification, Fig. 1)^7,8^. These natural history classifications draw from a breadth of understanding of reef development and ecology, but have sometimes lacked a standardised approach (e.g. compared to highly regulated knowledge structures such as taxonomic classification of organisms, linguistics or computer science^9^), due to difficulties of integrating diverse information sources and complex knowledge. Divergent geographic, linguistic and disciplinary lenses have occasionally caused divergence in a) nomenclature (terminology of reef features), b) structure (how some of these features are grouped) and c) meaning (how terms are interpreted and their relevance for practical research). For example, the term *Back Reef* can be interpreted in many ways^10^. *Back Reef* may be understood differently by an ecologist vs a geologist, or a Caribbean vs a Pacific-based researcher, or have a different relevance to a conservation practitioner vs ecosystem modeller.

In the last 40 years, technological advancements in the field of remote sensing have led to a different approach to reef classification^11^. Specifically for scaling habitat mapping from remote sensing, this largely computational approach (*Machine-led* classification, Fig. 1), involves the *a posteriori* seascape-scale grouping of pixels into classes, often using probability-based sampling of a one or two broad-coverage remote sensing datasets (e.g., spectral reflectance, bathymetry)^12^. Classification methods developed by remote sensing scientists have driven rapid expansion of reef mapping efforts from reef-scale to ocean-scale extents^13^. However, these approaches are known to be limited by the spatial, spectral, radiometric and temporal resolutions of source data used in the classifications^14^, and sometimes do not adequately consider the wealth of natural history, existing “traditional” coral reef classifications and associated terminology, or the requirements of users.

Computers are revolutionising our ability to classify multidimensional data sources and are allowing mapping and modelling at far greater scales than previously possible using expert-led approaches. However, careful consideration of machine-led classifications and how they align with traditional reef knowledge is vital in ensuring this expanding ability to map is still generating products that support the practical needs of the science and conservation community. Three challenges related to remote sensing classifications can hamper effective use of new generation coral reef maps.

1. The first challenge relates to divergence in how a natural scientist or manager understands a complex system, and how that system can be characterized by biophysical data layers (e.g., satellite imagery, physical and environmental data layers). A mismatch can undermine the relevance of the map product being created to potential users.
2. Secondly, a lack of *transparency* about classification processes is a prevalent problem in large scale mapping and can be a barrier to effective use of maps. A recent study found just 32% of publications provided sufficient information to replicate a series of mapping studies (99/304 published studies reviewed^15^). Lack of *transparency* may confound interpretation of classes by users.
3. The third challenge relates to communication: map classes are frequently not sufficiently defined or described (*clarity*) or made available to users (*accessibility*). A recent review found that only 52% of satellite-derived coral reef and seagrass habitat maps were accompanied by a detailed class descriptor that would allow users to interpret mapped classes (49/72 published coral reef and seagrass habitat maps^16^).

To address these challenges we have developed *Reef Cover*, a classification system consisting of 17 shallow tropical coral reef internal geomorphic class descriptors. Developed specifically for regional-to-global reef mapping to support science and conservation, classes are transferable between two domains: the traditional ecological-biophysical perspective and the earth observations systems view of reefs (Fig. 1). The classification aims to bridge disciplinary gaps by focussing on attributes of reef features that can be mapped from most remotely-sensed data (Methods: Step 2), while simultaneously aligning with foundational geo-ecological understanding of reef morphology (Methods: Step 1)^17^. Class definitions were developed with users in mind (Methods: Step 3), and accessibility issues were addressed by making *Reef Cover*’s detailed descriptors, user-friendly, simple, open access and freely available.

Finally, to demonstrate the application of *Reef Cover*, the classification was trialled in two mapping exercises (see Technical Validation). *Reef Cover*’s success was evaluated on how effectively it has been used to support creators of large-scale coral reef maps to generate products that have promoted management and conservation outcomes for reefs.

## Methods

*Reef Cover* was specifically developed to support the process used to produce and deliver globally applicable coral reef mapping products from remotely sensed data^16^. The typology acts as a key to bridge historic and contemporary knowledge, plot-scale and aerial viewpoints, and pixel data with natural history to convert pixel data into information in a form suitable for reef management decisions. Accompanying case-studies^18,19^ we use to demonstrate its application are also publicly available. The mapping products described in each case study were developed specifically using *Reef Cover* to support science and conservation of coral reef ecosystems.

We sought to develop a robust system which balances the geomorphic complexity of reefs with the need to develop high accuracy maps of each class in the system. The result is a 17-class system that can be (i) applied to remote sensing datasets for future mapping, (ii) used to interpret coral reef maps (iii) effectively disseminated to users – mainly in coral reef ecology and conservation space – in a way that promotes research and conservation.

Three steps were used in the development of the classification scheme.

1. Step 1. **Review**. Existing coral reef geomorphic classification schemes (expert-led classifications from Darwin’s 1842 reef classification^20^ to the *Millennium Coral Reef Mapping Project* classification^21^) were carefully reviewed^22^ to identify synergies in terminology and definitions for reef features, and evaluate how well common features can be described in terms of remote sensing biophysical data. The review allowed us to develop a set of classes that build constructively on previous foundational knowledge on coral reef geomorphology and are relatable to existing mapping and classification efforts, and addresses the challenge of *relevance*.
2. Step 2. **Development**. *Reef Cover* classes were then derived from attributes data, building on established machine-led reef mapping theory^13^. Physical attributes datasets commonly available to remote sensing scientists were examined to refine a set of 17 meaningful internal reef classes that relate to broader interpretation from a natural history point of view, gathered in Step 1. A workshop was organised to gather feedback on classes. Clarity around how each class relates to attribute data addresses the challenge of *transparency*.
3. Step 3. **Dissemination**. *Reef Cover* classes were then documented^23^ in a way to promote re-use and cross-walking, with a strong focus on needs of the users, to address the challenges of *clarity* and *accessibility*. Development of the *Reef Cover* document considers and details the 1) *relevance* e.g. rationale behind why it was important to map this class, but also broader global applicability of the class, 2) *simplicity* e.g., promoting user-uptake by employing plain language, not over-complicating descriptors and limiting the number of classes to manageable amount, 3) *transparency* supplying methodological basis behind each class, and exploring caveats and ambiguities in interpretation, 4) *accessibility* including discoverability, open access and language translations to support users, and 5) *flexibility* allowing for flexible use of the scheme depending on user needs, allowing for flexible interpretation of classes by providing cross-walk to other schemes and existing maps, and making the classification adaptable, and open to user feedback (versioning).

Finally, as a proof-of-concept the *Reef Cover* classification was tested in two large scale coral reef mapping exercises: one in the Great Barrier Reef ^24^ (Case Study 1) and one across Micronesia^19^ (Case Study 2, Technical Validation section). During this process, the *Reef Cover* dataset was reviewed to assess how useful it was for both a) producers using *Reef Cover* to map large coral reef areas from satellite data, and b) consumers using *Reef Cover* to interpret map products for application to real world problems.

### Step 1. Review. Building global classes on foundational reef mapping and classification work

#### Global reef mapping: the need for a geomorphic classification to map coral reefs at scale

Coral reefs represent pockets of biodiversity that are widely dispersed, often remote/inaccessible and globally threatened^2,3^. Communities and economies are highly dependent on the ecosystem services they provide^25–27^. This combination of vulnerability, value and a broad and dispersed global distribution mean global strategies are needed for reef conservation, for which maps (and the classifications that underpin them) play a supporting role. Global coral reef maps have been fundamental to geo-political resource mapping and understanding inequalities^28,29^, the valuation of reef ecosystem services^26^, understanding the past^30^, present^31^, and future threats to reefs^32^, supporting more effective conservation^33,34^ and reef restoration strategies^35,36^, and facilitating scientific collaborations and research outcomes^37^. Reef conservation science and practice may particularly benefit from technological advancements that allow delivery of more appropriate map-based information, particularly across broader, more detailed spatial scales and in a consistent manner^34,36,38^.

#### Existing expert-led reef classifications

Traditionally, coral reef features have been grouped based on observations of morphological structure, distributions of biota and theories on reef development, gleaned from aerial imagery, bathymetric surveys, geological cores and biological field censuses by natural scientists^7,39^ (Fig. 1). Natural scientists were struck by both the uniformity and predictability of much of the large-scale three-dimensional geomorphic structure of reefs and biological partitioning across that structure, and how consistent these characteristic geologic and ecological zones were across large biogeographic regions^20,40^). Technological developments of the 20^th^ century, such as SCUBA demand regulators and compressed air tanks (commercially available in the 1940s^41^), acoustic imaging for determining seafloor bathymetry (e.g., side-scan sonar developed in the 1950s), light aircraft for aerial photography (first applied in the 1950s^42^) and lightweight submersible drilling rigs for coring (applied in the 1970s^43^), allowed reef structure to be viewed from fresh perspectives. New aerial, underwater and internal assessments of reef structure expanded the diversity of external and internal classes, with hundreds of new terms for features defined^7,8,10^ (Online-Only Table 1). However, the localised nature of most of these applications (Fig. 1) meant that many of the classes developed using these tools were region-specific, leading to experts warning against too heavy a reliance on “*the imperfect and perhaps biased existing field knowledge on reefs*” for developing global classifications^44^.

#### Existing reef classifications derived from satellite data

Shallow water tropical coral reefs are particularly amenable to global mapping from above^12^. They develop in clear, oligotrophic tropical waters, so many features are detectable from space^45^. Satellite technology has spawned a wealth of data on reefs, enabling large area coverage, with resolution of within reef variations. Initial approaches to reef mapping in the 1980s expanded our traditional viewpoint from single reef mapping and extent mapping to detailed habitat mapping of whole reef systems^46^. Through the 1990s and early 2000s evolving field survey techniques described above enabled more effective linkage of ecological surveys to remote sensing data^47,48^. Accessibility to higher spatial resolution images over larger areas in combination with detailed field data, physical attributes and object-based analysis resulted in large reef area mapping^13,21,49,50^. In the last five years, the increase in daily to weekly global coverage of this type of imagery, in combination with cloud-based processing capability has expanded to a global capability for reef mapping^38^. This is a new type of global information that requires a different approach to classification to make sense of complex natural systems at ocean scales.

One of the first steps in creating the *Reef Cover* classification was reconciling existing classification schemes across the nomenclature driven by disciplinary, linguistic and regional biases. To do this we conducted a review of reef geomorphic classifications, looking for consistencies and usage of terms that transcended divides in discipline^22^ (see summary in Online-Only Table 1).

#### Scaling and consistency: choosing an appropriate level for Reef Cover

Remote sensing scientists have been developing automated methods to make sense of the increasing availability of earth observation data over coral reefs, yielding information on ecosystem zones derived from data sources such as spectral reflectance and bathymetry at increasingly larger scales^12,51^. As more data increasingly reveal the diversity and complexity of reefs, selecting an appropriate level at which to map reefs on the global scale requires balancing the need for a limited number of classes that can be mapped consistently based on available earth observation data, with user need for comparable information.

##### Reef type classification

Morphological diversity can make global geomorphic classification – particularly between reefs (at the “reef type” level, e.g., *Fringing*, *Atoll* reefs) - challenging. Divergent regional morphologies (e.g., Pacific atolls *vs* Caribbean fringing reefs) and endemic local features (e.g., Bahamian shallow carbonate banks, Maldivian farus) are created by underlying tectonics, antecedent topography, eustatics, climate and reef accretion rates which can all vary geographically^52^. The diversity of reef types is reflected in the large number of classes defined in the impressive *Millennium Coral Reef Mapping Project* (68 classes at the between-reef geomorphic level L3), the most comprehensive globally applicable coral reef classification system to date^21^.

##### Geomorphic zone classification

Internally, reef morphology becomes a lot more consistent. Physical boundaries in the depth, slope angle and exposure of the reef surface create partitioning into “geomorphic zones” (e.g., *Reef Flat*, *Reef Crest*), developed in parallel to the reef edge and coastlines and generally with a distinct ecology^17,39,53^. These internal patterns of three-dimensional geomorphic structure can be remarkably predictable, even between oceans. This makes geomorphic zonation a good basis for consistent and comparable mapping at regional to global scales^54^. Moreover, congruence between geomorphic zones and ecological partitioning means that ecological understanding can be derived from geomorphic habitat classes, making geomorphic mapping valuable to conservation practitioners^55^.

##### Benthic classification

Many classifications developed for reef mapping (e.g., Living Oceans Foundation^49^, NOAA Biogeography Reef Mapping Program^50^), monitoring (Atlantic and Gulf Rapid Reef Assessment^56^, Reef Cover Classification System^57^, Reef Check^58,59^) and management (Marine Ecosystem Benthic Classification^60^) have included an ecological component. Classifying reef benthos is important as associated metrics, such as abundance of living coral and algae, are widely used indicators of ecosystem change. However, most classifications that consider benthic cover are operational at reef^61^ to regional scales, due to the need for very high-resolution remote sensing data^11^ (e.g. from UAVs and CASI^62^, or high-resolution satellites like QuickBird and WorldView 1 m) to be able to reliably determine classes such as coral cover and type, soft coral, turf, coralline algae, rubble and sand. A comprehensive benthic coral reef classification^23^ that met the Reef Cover objective of being globally scalable (both in terms of remote sensing biophysical data availability and processing capabilities) but that also fully recognises and includes the rich benthic detail required to address ecological questions at sub-metre scales is beyond the scope of this classification. In the coming years it is likely that further advancements in technology – both downscaling of remote-sensing and up-scaling of field observations^63^ - will enable us to address this spatial mismatch.

The challenge of creating the *Reef Cover* classification was to create a set of classes that related to natural science observations, despite using data pulled from remote sensing. Intra-reef zones defined by natural scientists often represent different biophysical /ecological communities that in turn reflect environmental gradients (e.g., in light, water flow) and geo-ecological processes (sediment deposition, reef vertical accretion) below the water that led to the arrangement^17^. However, these classes frequently also can be related to biophysical information on slope, depth and aspect that can be determined remotely. A thoughtfully prepared classification – that adheres to Stoddart’s (1978) classification principles, which state that classes should be explicit, unique, comprehensible, and should follow the language of prior schemes - can support production of maps and other science (monitoring, management) that are still relevant to historic work but that can go forward with consistent definitions^21^.

### Step 2. Development. Creator requirements - relating *Reef Cover* classes to remote sensing data

Development of appropriate mapping classes requires a sensitive trade-off between the needs of users (in terms of the level of detail needed, appropriate for scaling, consistent across regions, simple enough to be manageable but detailed enough to be understandable), and the input data available and quality of the globally repeatable mapping methods of the map producers.

While vast in terms of scalability, data producers are more constrained in terms of sensor capabilities such as spatial resolution (limited to pixels) and depth detection limits (limited by light penetration), and processing power (high numbers of map classes becoming more computationally expensive). Physical conditions and colour derived from remote sensing, along with their textural and spatial relationships, can be linked to reef zonation^13^, with depth and wave exposure being particularly important information to explaining geomorphology^64^.

To select a set of *Reef Cover* classes that could be defined by attributes available from most commonly available public access or commercial satellite data, but that also corresponded to common classes found in the classification literature, and relevant from a user perspective, we looked for intersectionality between physical attribute data that can be derived from satellites but also help shape and define reef morphology.

#### Physical attributes

**Fig. 2:**
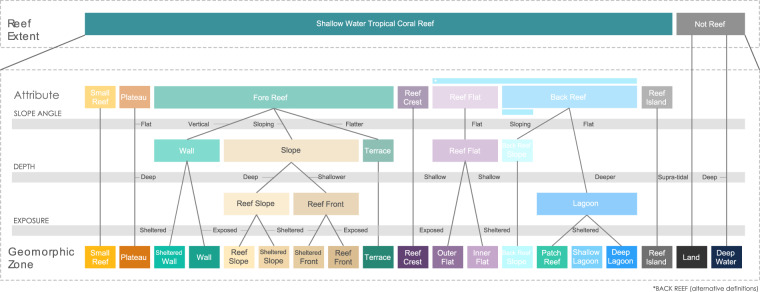
Physical attributes derived from remote sensing data such as depth, slope angle and exposure are sufficient to delineate some of the key geomorphic reef zones in the classic literature. The coral reef classifier for global scale analyses of shallow water tropical coral reefs shows how relative measures can characterise reef zones.

The physical environment – light, waves and depth – plays a deterministic role in reef structural development and the ecological patterning across zones^39^. Underlying geomorphic structural features can almost always be characterised in terms of three core characteristics: i) depth, ii) slope angle and iii) exposure to waves (Fig. 2).

##### Depth

Depth is a useful attribute for bridging human and machine-led classifications. Bathymetry can be derived from spectral information from satellites since the absorption of light at specific wavelengths also has known relationships with water column depth^65^ but also relates to reef geomorphology (due to role of primary production in powering biogenic calcification)^66^. Bathymetric data also provides the basis for other critical depth-derived products, slope and aspect, which are used to distinguish geomorphic classes and reef environmental parameters, e.g., exposure to breaking wave energy (Fig. 2).

*Reef Crest*, for example, is often described as the shallowest part of the reef ^62^, while *Lagoon* represents a deep depression in the reef structure^57,67^. Depth thresholds are sometimes defined: a threshold of 10 m was suggested to differentiate true lagoons from shallow water areas^68^, and an 18 m threshold has been used to distinguish *Reef Front* from *Reef Slope*^17^. In the *Reef Cover* classification, depth was particularly important for distinguishing Fore Reef classes (e.g. *Reef Slope*, *Terrace*) from *Reef Crest* and *Reef Flat* classes (Table 1, Fig. 2). Generally, tides and variability in water clarity and regional eustatic discrepancies in reef top depth (e.g., *Reef Flat* in Atlantic systems generally lie much lower with respect to tides than in the Indo-Pacific^67^) mean relative depths are more appropriate, which is why absolute numbers were not used in *Reef Cove*r definitions.

**Table 1:** Attributes of reef zones that help support classification.

ATTRIBUTE		Depth					Slope				Exposure			Substrate			Colour			Rugosity			Benthic Cover			
REEF COVER CLASS		Supratidal	Intertidal	Subtidal			Horizontal	Shallow	Steep	Vertical	Exposed	Average	Sheltered	Hardbottom	Mixed	Soft substrate	Bright	Medium	Darker	Low	Medium	Complex	Coral	Coralline	Sandy	Seagrass
				Shallow	Medium	Deep																				
**Reef Slope**	**Fore Reef**				✓	✓		✓	✓		✓			✓				✓	✓		✓	✓	✓			
**Sheltered Slope**					✓	✓		✓	✓			✓		✓				✓	✓			✓	✓			
**Reef Front**				✓	✓			✓	✓		✓			✓					✓			✓	✓	✓		
**Sheltered Front**				✓	✓			✓	✓			✓		✓					✓			✓	✓			
**Wall**					✓	✓				✓	✓			✓							✓	✓	✓	✓		
**Sheltered Wall**					✓	✓				✓		✓		✓							✓		✓			
**Terrace**					✓	✓	✓				✓	✓		✓					✓		✓	✓	✓		✓	
**Reef Crest**			✓	✓	✓		✓	✓			✓			✓			✓	✓						✓		
**Outer Reef Flat**	**Flat**		✓	✓			✓				✓	✓		✓			✓				✓	✓	✓	✓		
**Inner Reef Flat**				✓	✓		✓	✓				✓			✓		✓				✓		✓		✓	
**Lagoon**	**Back Reef**					✓	✓	✓					✓			✓	✓	✓		✓					✓	
**Patch Reef**				✓	✓		✓	✓					✓	✓				✓					✓			
**Shallow Lagoon**				✓	✓		✓	✓					✓			✓	✓			✓					✓	
**Back Reef Slope**					✓	✓		✓					✓		✓	✓		✓			✓		✓		✓	
**Plateau**	**Other**				✓	✓	✓	✓				✓		✓					✓		✓		✓			
**Small Reef**					✓	✓					✓	✓		✓	✓			✓	✓		✓		✓			
**Reef Island**		✓	✓												✓				✓							
**Deep Water**	**Not reef**					✓													✓							
**Land**		✓												✓	✓	✓			✓							

Physical and biological zonation are often closely linked to an array of gradients in depth, wave action, current, light and sediment on different parts of the reef. These physical attributes can be used to define meaningful ecological characteristics of geomorphic zones. The attributes listed can be both derived from remote-sensing data and align with knowledge about reef natural history, and so are useful in helping distinguish 17 classes based on depth, slope, exposure, colour and texture attributes. Rugosity and benthic cover are attributes that will become more important in determining classes as satellite resolution continues to improve at large scales.

##### Slope

Slope angle, either absolute angle or discontinuities in angle acting as a break between zones, is an important differentiator of reef zones. *Reef Flat*s are defined as being horizontal ‘flattened”^69^ “flat-topped”^70^; *Fore Reef* slope zones often include references to slope angle (e.g., in one classification *Fore Reef* has been defined as “any area of the reef with an incline of between 0 and 45 degrees”^62^), and *Walls*– common on atolls - are “near vertical” features. Variability in slope continuity can also be an important way to demarcate zones^71^. Montaggioni illustrated a range of representative profiles across atolls and barrier reefs, with convoluted profiles allowing subdivisions of reef slope, particularly across fringing reefs which are less likely to show a uniform reef slope than an atoll^53^, and *Reef Crest* is sometimes defined as a demarcation point separating the *Fore Reef* from the *Reef Flat*^53,62,72^. Where water depth can be derived from remotely sensed spectral data, bathymetry can be used to directly calculate slope (i.e., by calculating the slope angle between a pixel and its neighbours) or by considering the local variance in depth (e.g., the standard deviation in depth values within some radius of each pixel).

In the *Reef Cover* classification, slope angle data were used to distinguishing *Fore Reef* classes such as *Reef Slope* and *Reef Front*, from horizontal classes such as *Outer and Inner Reef Flat* and *Lagoons* (Table 1, Fig. 2).

##### Exposure

Physical exposure of reefs is a key driver of zonation. *Reef Crests* – linked to wave breaking – are often described as “an area of maximum wave shoaling”, i.e. a zone that absorbs the greatest wave energy^62,69^. *Fore Reefs* are frequently sub-divided based on relative exposure (e.g. exposed vs sheltered slope, or windward vs leeward^57^). Exposure influences profile shape and importantly the communities growing in the zone, so that slopes with identical profiles could have very different communities^57,73^. Sometimes these zones are related to the communities found there. Meanwhile, exposure across the reef means back-reef zones contain sheltered water bodies. Together with data on water depth and bathymetry, wave energy data was key for distinguishing key *Reef Cover* classes^74,75^.

##### Colour and texture

**Fig. 3:**
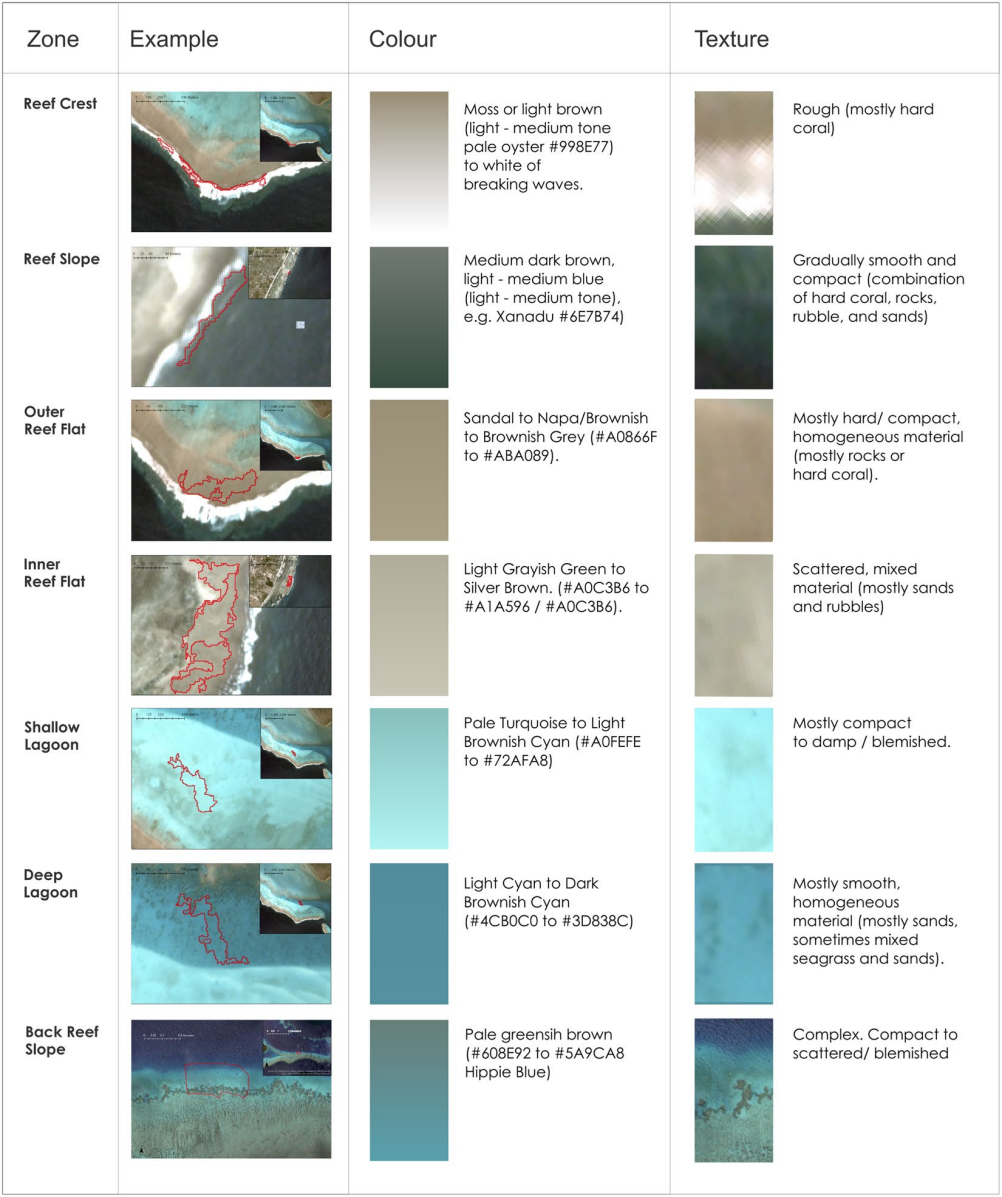
Satellite-derived colour and texture can be informative in distinguishing *Reef Cover* classes of relevance to ecologists and managers, since spectral reflectance mirrors the benthos which in depositional areas may be dominated by reef-derived sediments, or on hard substrate may reflect benthic communities. Not all zones can be distinguished by colour alone (e.g., walls and steep slopes), but examples of zones with clear colour/texture differences are outlined in red.

Sub-surface spectral reflectance data can provide measurements of reef colour and texture over large areas. Concentrations of photosynthetic pigments in coral, algae and seagrass as well as light scattering by inorganic materials means spectral reflectance can also be used to determine biophysical properties of the reef ^65^. Colour and texture information derived from satellites can be used to manually draw polygons around similar geomorphologic units or habitats but provide the basis to drive image-based thematic mapping (such as digital number, radiance, reflectance) and texture, through spectral processing^64^. Texture measures are also used to improve classification by allowing spectrally similar substrates like corals and macroalgae to be distinguished. *Reef Flats*, for example, having a single driver of zonation, in contrast to several drivers on most other zones, makes benthic zonation particularly distinct^39^, and easily detectable as coloured bands in aerial images of reef flats. This allows colour and texture to be used to distinguish *Outer Reef Flats*, which have a greater component of photosynthetically active corals and algae, from *Inner Reef Flats* which appear brighter due to a higher proportion of sand build up in this depositional area (Fig. 3).

#### Spatial relationships

##### Size and shape

The size and shape of reef features can help determine *Reef Cover* class. Many large-scale reef structural features appear elongate as the shelf constrains shape – and reef morphology can even help predict shape as they constrain accommodation space and influence deposition^76^. *Reef Flats*, for example, boast the broadest horizontal extent of any geomorphic zone, typically 500 to 1000 m across, but reaching several kilometres in width across some Pacific atolls^71^. *Lagoons* also tend to be broad in width although width and shape can be variable depending on reef type. Understanding some of these characteristics can help determine classes, although these are usually defined relationally rather than by application of size thresholds.

##### Neighbourhood and enclosure

Natural scientists agree that reefs feature three major geomorphic elements: a *Fore Reef*, a *Reef Crest* and a *Back Reef* (although subdivisions and complexities exist around these). Because of the influence of large-scale processes on reef development, these zones occur in order^17,39,53^. *Reef Crest* is arguably the most defining characteristic of any reef – the break point at which a sharply defined edge divides the shallower platform from a more steeply shelving reef front^71^, around which other geomorphic zones arranged in parallel^77^. As a result, spatial arrangement of zones can be informative for mapping (Table 2). For example, *Back Reef* is often defined as being contiguous to the *Reef Crest* (*Back Reef* is often defined as any reef feature found landward of the crest).

**Table 2:** Relational characteristics of Reef Cover classes displaying neighbourhood (including adjacency and enclosure rules) used to distinguish internal coral reef geomorphological zones.

ADJACENCY RULES	Reef Slope	Sheltered Slope	Reef Front	Sheltered Front	Wall	Sheltered Wall	Terrace	Reef Crest	Outer Reef Flat	Inner Reef Flat	Deep Lagoon	Patch Reef	Shallow Lagoon	Back Reef Slope	Plateau	Small Reef	Reef Island	Deep Water	Land
Reef Slope	✓	—	✓		—	—		✓										✓	
Sheltered Slope	—	✓		✓	—	—		✓					—	✓				✓	
Reef Front	✓		✓		✓			✓							—			✓	
Sheltered Front	—	✓		✓	✓			✓					—	✓	—			✓	
Wall	✓				✓	✓		✓							—			✓	
Sheltered Wall		—			✓	✓		✓					—	✓	—			✓	
Terrace	—	—			✓	✓	✓	✓						✓	—			✓	
Reef Crest	✓	✓						✓	✓	—									
Outer Reef Flat	—	—						✓	✓	✓	—		—	—			—		
Inner Reef Flat	—	—							✓	✓	—	—	✓	—			✓		—
Deep Lagoon									✓	—	✓	✓	✓	—			—		—
Patch Reef										—	✓	✓	✓	✓					
Shallow Lagoon									—	✓	✓	✓	✓	✓			✓		✓
Back Reef Slope									—	✓	✓	✓	✓	✓				—	
Plateau			—	—	—	—	—								✓	—		✓	
Small Reef															—	✓		✓	
Reef Island									—	✓	—		✓	✓			✓		
Deep Water	✓	✓	✓	✓	✓	✓	✓	✓						—	—	✓		✓	
Land									—	✓			✓	✓					✓
**ENCLOSED BY RULES**	**Reef Slope**	**Sheltered Slope**	**Reef Front**	**Sheltered Front**	**Wall**	**Sheltered Wall**	**Terrace**	**Reef Crest**	**Outer Reef Flat**	**Inner Reef Flat**	**Deep Lagoon**	**Patch Reef**	**Shallow Lagoon**	**Back Reef Slope**	**Plateau**	**Small Reef**	**Reef Island**	**Deep Water**	**Land**
Reef Slope	n/a		—		—		—											—	
Sheltered Slope		n/a		—		—	—											—	
Reef Front			n/a															—	
Sheltered Front				n/a														—	
Wall					n/a													—	
Sheltered Wall						n/a													
Terrace							n/a												
Reef Crest	—	—						n/a											
Outer Reef Flat								—	n/a										
Inner Reef Flat									—	n/a									
Deep Lagoon								—	✓	✓	n/a		✓	✓					
Patch Reef										—	✓	n/a	✓	✓					
Shallow Lagoon								✓	✓	—			n/a	✓					
Back Reef Slope										✓				n/a					
Plateau											—				n/a			✓	
Small Reef																n/a		✓	
Reef Island	—	—			—	—				✓	—		✓	✓			n/a	—	
Deep Water																		n/a	
Land	—									—			—	—					n/a

Blank cell = not usually neighbours/enclosed by; Yellow = May sometimes be enclosed by/neighbours; Green = typically enclosed by/neighbours. (To interpret the table move from Column 1 to row 1 (e.g. “is Small Reef enclosed by Deep Water?”).

Enclosure to semi-enclosure within a bordering reef construction (e.g., in lagoons^68^) is another feature used classically to define reef zones, but that could also be derived from satellite imagery.

**Fig. 4:**
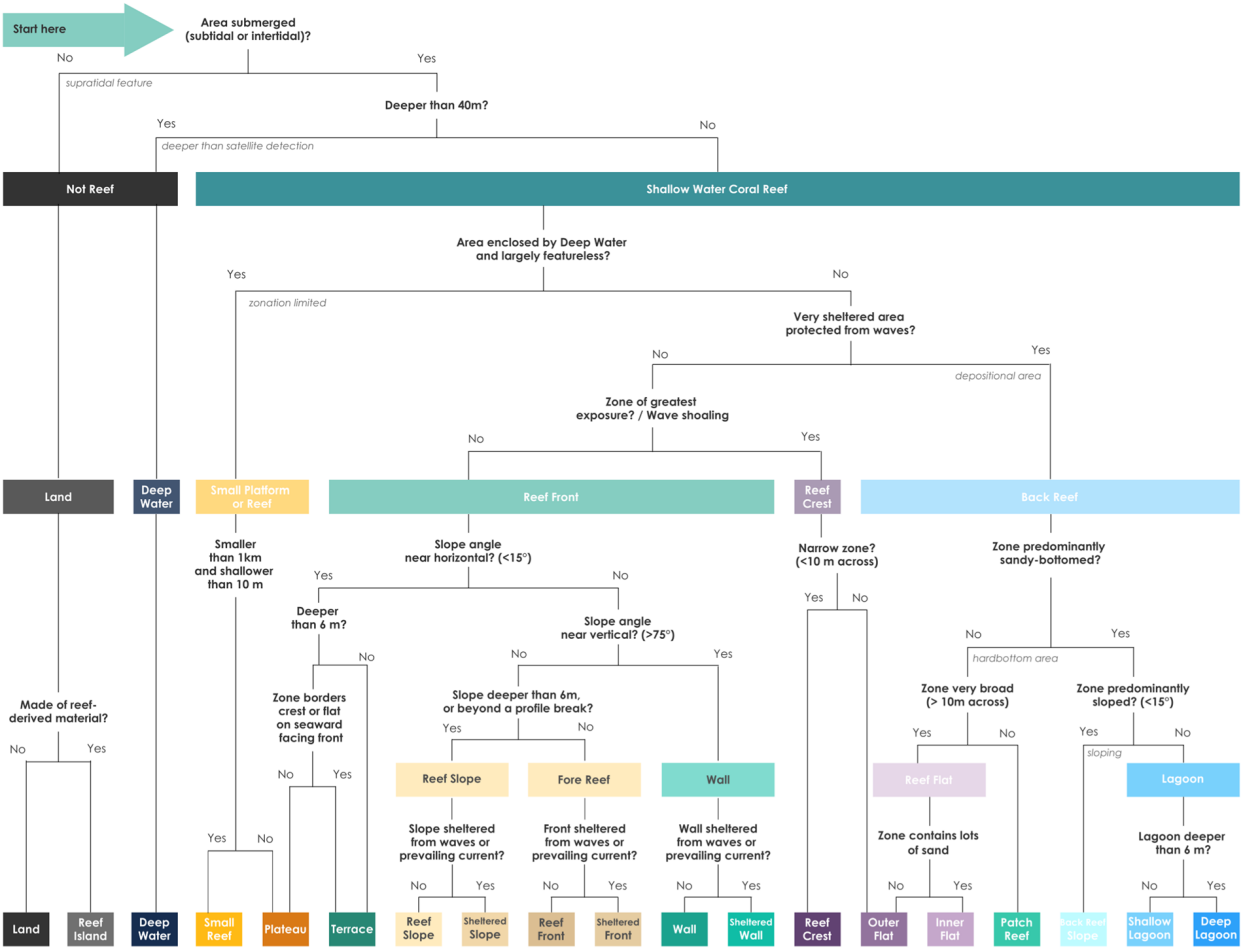
Example decision tree for classification of intra-reef zonation using *Reef Cover*. The decision tree for use by mappers is based on information that would typically be available at the global scale, and related to the physical attributes (depth, slope angle and exposure), colour and texture, and spatial relationships. Here a mix of *a priori* logical or philosophical grounds taken from a review of literature, tailored to fit a methodology limited by the data.

The *Reef Cover* typology presented is derived from earth observation data, but attempts to link classes to genetic process, social, ecological and geological importance^23^. By focussing on the attributes of depth, light, exposure, colour and texture and spatial relationships that are common to both domains, our traditional biophysical knowledge of reefs can be integrated with remote-sensing capabilities. Attributes can be combined to make decision trees (Fig. 4) to help use satellite data to map reefs at the global scale. The *Reef Cover* list of classes can all be distinguished from these physical attributes alone, supporting production of maps that are still relevant to existing work but that can allow computationally inexpensive determination of mapping classes to beyond what was previously possible^38^.

### Step 3. Dissemination. Providing user friendly *Reef Cover* class descriptors that facilitate uptake and use

Computers have revolutionised our ability to classify multidimensional data sources, which allows mapping and modelling at far larger scales for the same effort compared to a human taxonomist. However, without proper consideration of the needs of the end user, classified data may not be effectively applied to conservation challenges^4^. The *Reef Cover* classification was developed with five user-needs in mind: relevance, simplicity, transparency, accessibility and flexibility.

#### Relevance

Different habitats within reefs contribute differently to biological and physical processes. For example, *Reef Crests* play a disproportionate role in coastal protection, dissipating on average 85% of the incoming wave energy and 70% of the swell energy^78,79^; *Reef Slopes* supply an order of magnitude more material to maintain island stability^61,80^; shallow *Reef Front* areas often host more coral biodiversity^81^; *Reef Flats* support herbivorous fish biomass^82^ and accessibility of *Lagoons* often affords them cultural importance as places important for artisanal harvesting^83^. A classification that effectively captures the appropriate diversity of these habitats can therefore better inform social, biological and physical studies^36^, such as global conservation planning to safeguard reefs, for example, in order to meet the *Convention on Biodiversity* Aichi targets^34^. Map classes need to reflect differences of interest to a wide range of reef scientists, from oceanographers to paleoecologists and fisheries scientists – so careful consideration of natural history is important. Global mapping is usually to enable spatial comparisons, so a classification that is globally applicable was also important.

To explore relevancy, a crosswalk was performed between *Reef Cover* and a selection of major regional to global coral reef classification^10,60,84^, mapping^22,36,85^ and monitoring efforts^7,8,56^, to make sure important classes from established classifications had not been missed^23^ (Online-Only Table 2).

#### Simplicity

Simplicity was achieved by (1) choosing an appropriate mapping scale (internal geomorphic classes), (2) limiting the number of geomorphic classes (17 classes), and 3) providing clear (1 line) descriptors with additional information to address issues of semantic interoperability.

1. *Reef Cover* was developed to provide consistent mapping of reefs across very large areas: classification of geologic and ecological zones is much more amenable to mapping using remote sensing, given greater consistency in geomorphology across large biogeographic regions^32^. Satellite data has supported the development of several detailed regional “reef type” classifications, such as nine reef classes for the Great Barrier Reef from Landsat imagery^73^, six reef classes from the Torres Straight^74^ and 16 classes for the Red Sea from Quickbird^6^. However, local reef type classifications are not always applicable globally due to large regional discrepancies in Reef Type. As a result, detailed reef type typologies are more suitable for local to regional classifications^6,35^. For global mapping, an internal geomorphic approach is better. Finer spatial scale classifications from satellite data are also challenging, due to differences in the spatial scale at which spectral data can be generated (metres) and which benthic assemblages display heterogeneity (sub-metres)^32^. Medium spatial resolution multispectral data (5 to 30 m) is the most commonly used satellite information used for coral reef habitat mapping^42^, and classification of internal geomorphic structures may be best suited to this kind of data.
2. Reviews of habitat mapping from remote sensing found the number of map classes averages 18 at continental and global scales^13^. More than this can become overwhelming for users and computationally expensive for developers at this point in time. Many coral reef classifications contain four or five hierarchical levels and high numbers of classes: the *Millennium Coral Reef Mapping Project (MCRMP*) was ambitious in developing a standardised typology that captured much of the reef type diversity, but despite defining over 800 reef classes defined at the finest (level 5, essential for local reef mapping) scale^36^, level 3 (68 classes) continue to be more popularly adopted in publications using this dataset. To keep the classification simple, *Reef Cover* was limited to 17 geomorphic classes, with simple one line definition provided. A limited class was needed 1) to make it manageable for users, 2) to make it computationally manageable for very large (regional and global) data processing and 3) reduction in classes compared to MCRMP allowed for consistent automated mapping at the global scale – so that whole regions could be directly compared for monitoring and management.
3. Short definitions were provided in plain language for simplicity. To address additional uses issues of semantic interoperability each *Reef Cover* class definition also outlines other commonly used terms for concepts (synonymy) and explains different interpretations of the same meanings and understanding of the relations between concepts.

#### Transparency

One barrier to the use of analysing and interpreting big data is user-friendliness. Of 79 coral reef mapping attempts reviewed (62 benthic coral reef maps, 6 geomorphic coral reef maps and 11 mixed), only 13% were accompanied by a clear classification that defined the meaning of map classes^14^. Describing how the classification relates to data (Step 2) and producing a detailed descriptor (Step 3) along with a diagram allows classification to be understood and also adopted for different projects. We also attempted to address transparency by relating *Reef Cover* classes to other major global mapping and monitoring efforts (Online-Only Table 2) and providing a decision support tree for users (Fig. 4)^69^.

#### Accessibility

Another barrier to the use of analysing and interpreting big data is access^75^. Much information remains locked behind paywalls, and additional barriers exist including discoverability. To promote accessibility and encourage use, all data were made publicly available (see Data Records section for access). Terms were translated into different languages, as science published in just one language has been shown to hinder knowledge transfer and new findings getting through to practitioners in the field^86^.

#### Flexibility

One criticism of thematic habitat maps derived from remote sensing is a lack of flexibility: categorical descriptions of habitats are by design a discrete simplification of the ecological continua, thus classifications limit the interpretation and questions that can be asked^76^. Flexibility issues were addressed by 1) not prescribing absolute thresholds to each class, instead providing information on how classes relate to each other (Tables 1–3) allowing a) map producers to adapt application of *Reef Cover* to their own needs, and b) users to interpret with flexibility, 2) providing additional information (Standard Descriptors) including main features, exceptions to rules and broadness as to provide users with a broader understanding of hidden complexities when interpreting class meaning, 3) remaining open to feedback, we hope this *Reef Cover* version 1 can be improved upon with feedback from the community.

**Table 3:** Table detailing how *Reef Cover* classes were used in each Case Study, and confidence of producers in determining each class (scored from 1 to 10, with 1 being very low confidence and 10 being very high) from satellite information in Case Study 2.

*Reef Cover* classes	Case Study 1. (15 classes)	Case Study 2 (12 classes)	Class confidence	SD (eight experts)
Reef Slope	Deep Slope 10 m + Windward	*not used*	*not assessed*	
Sheltered Slope	Deep Slope 10 m + Leeward	*not used*	*not assessed*	
Reef Front	Slope 3–10 m Windward	Reef Slope	7.8	1.1
Sheltered Front	Slope 3–10 m Leeward	Sheltered Slope	6.8	1.5
Terrace	Plateau 3–10 m	*not used*	*not assessed*	
Wall	*not used*	*not used*	*not assessed*	
Sheltered Wall	*not used*	*not used*	*not assessed*	
Reef Crest	Reef Crest	Reef Crest	8.0	1.3
Outer Reef Flat	Outer Reef Flat	Outer Reef Flat	7.4	1.1
Inner Reef Flat	Inner Reef Flat	Inner Reef Flat	6.6	1.3
Back Reef Slope	Open Complex Lagoon	Back Reef Slope	5.9	1.7
Shallow Lagoon	Shallow Lagoon	Shallow Lagoon	7.2	1.8
Lagoon	Deep Lagoon	Deep Lagoon	8.0	0.9
Plateau	Plateau 10 m +	Plateau	6.8	1.4
Patch Reef	Patch Reefs	Patch Reef	7.0	1.5
Small Reef	Small Reef	Small Reef	7.2	1.6
Reef Island	Land	*not used*	*not assessed*	
		Terrestrial Flat	6.7	1.4

## Data Records

*Reef Cover* classification system (Version 1.0) presented in this paper has been made publicly available as a list of map classes and descriptors “*Reef Cover Classification (v1). Internal coral reef class descriptors for global coral reef habitat mapping*” (pdf format) through Dryad^23^.

The classification system includes 17 Reef Classes (Fig. 2) and their descriptors, including Standard *Name* - short class name, *Standard Label* - longer class name, *Standard Description* – detailed class descriptor, including context and main attributes (highlighted), *Translations* - Standard Name in different languages, *Synonyms* - list of commonly used synonyms.

Diagrams of how each class relates to major reef types, and a Glossary of terms is also provided.

The *Reef Cover Classification* document also contains an Attribute Table (Table 1), showing how classes relate to each other based upon on *Depth*, *Slope*, *Exposure*, *Substrate*, *Colour*, *Rugosity* and *Benthic Cover* data, information that might be available to mappers either from spectral reflectance, bathymetric, oceanographic or ecological datasets, and a Crosswalk Table (Online-Only Table 2) comparing how *Reef Cover* classes align with other major reef mapping and monitoring efforts.

### Additional supporting materials

Two additional resources accompany this data record in order to improve the uptake and use of *Reef Cover* classification. The first provides additional resources to support producers (remote-sensing data scientists) to create their own coral reef maps from *Reef Cover* (Methods)^87^. The second supplies users (coral reef practitioners and scientists) with downloadable regional case study maps developed using the *Reef Cover* classification (Maps)^19,24^ and presented as case studies in the Technical Validation section.

- **Methods**. Methods and code for use in global coral reef habitat mapping are freely available in Zenodo [10.5281/zenodo.3833246]^87^.
- **Maps**. Static coral reef habitat maps of the Cairns Management Region of the Great Barrier Reef Marine Park (GBRMP) produced using the *Reef Cover* classification are available on Pangaea [10.1594/PANGAEA.925657]^24^. Caroline and Mariana Islands produced using the *Reef Cover* classification outlined are available to download from Zenodo [10.5281/zenodo.3953052]^19^. Updates to the case study, along with maps for the entire globe using *Reef Cover* will be available in future [https://allencoralatlas.org/atlas/]^18^. Free online lessons to support users and creators of maps are also available [https://reefresilience.org/online/].

## Technical Validation

The goal of the *Reef Cover* classification is to facilitate conversion of large amounts of remote-sensing data into large-scale coral reef mapping products that support the work of coral reef practitioners and scientists. To validate *Reef Cover*, the classification was applied to two independent large-scale mapping efforts to test whether *Reef Cover* helped creators and users of the maps. The first, led by researchers in Australia, aimed to map coastal shelf reefs in a section of the Great Barrier Reef from 15 m pansharpened Landsat 8 data using an Object-based approach. Maps were specifically developed to support *Great Barrier Reef Marine Park* monitoring and management (case study #1). The second was an international collaboration to map two remote oceanic atoll chains in Micronesia as part of a global mapping effort to improve conservation outcomes for reefs, and used 5 m Planet Dove data and an automated cloud-based mapping approach (*Allen Coral Atlas* case study #2). Each map represents a significant leap forward in scientists’ ability to capture very large areas (thousands of kilometres) of shallow reef habitat in detail in a consistent and repeatable manner (see “Application” section). They also represent improvements in usability of maps: for the two case studies, we detail the challenges for producers in adopting the classification (see “Assessment” section), and successes were assessed on whether products could potentially be translated into real world scientific and conservation outcomes (see “Outcomes” section).

### Application: using *Reef Cover* to produce coral reef maps

#### Case Study #1. Large scale geomorphic mapping on the Great Barrier Reef for management

**Fig. 5:**
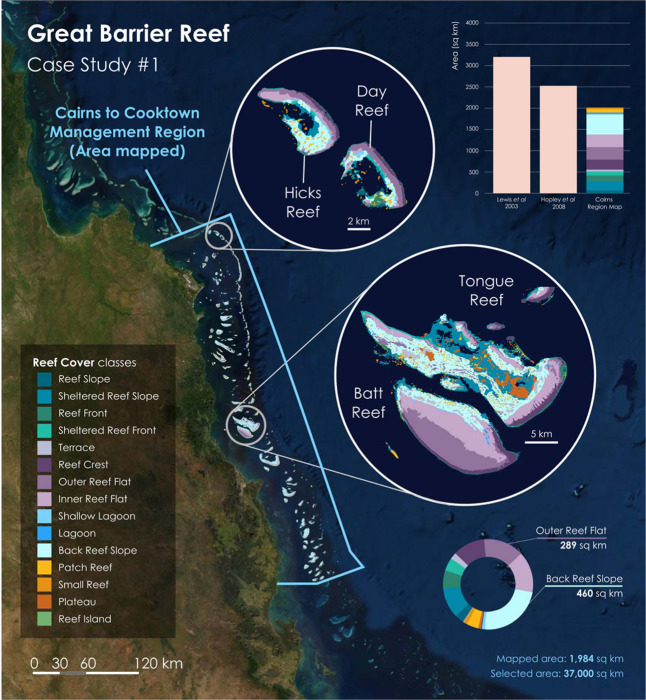
In Case Study 1, the *Reef Cover* geomorphic classification scheme was applied to an coral reef mapping exercise on the Great Barrier Reef, where 1,900 km^2^ of reef habitat was mapped from Landsat data from across the *Cairns Management Area* (37,000 km^2^) to support national management. *Reef Cover classes* (15/17 classes used) were foundational in the creation of the largest geomorphic map of the Great Barrier Reef to date, which allowed refined estimates of reef extent (barchart) as well as new estimates of coral habitat to be made (see Roelfsema *et al*. 2020 for details^36^).

In the first case study, *Reef Cover* was applied by a university-based research group to map 237 reefs in the central Great Barrier Reef Marine Park across a 37,000 km^2^ area to support local management efforts by government (Fig. 5).

The *Cairns Management Region* represents 6% of the Great Barrier Reef (GBR) but includes the tourist hubs of Cairns and Port Douglas, making it one of the most highly visited regions of the GBR. The Management Region supports more tourism than any other area as well as cultural activities, shipping, research, commercial and recreational fishing. The platform and ribbon reefs that occur in this region are typical of those on the Queensland Shelf, but very different to the oceanic atolls of case study #2. The high level of use of these reefs makes managing impacts in this area a priority for the Great Barrier Reef Marine Park Authority (GBRMPA), yet only outline extent maps of the large platform and ribbon reefs that make up this section existed previously^36,88^.

In this case study, the *Reef Cover* classification was used to guide the production of geomorphic coral reef habitat maps using object-based analysis (via Trimble eCognition 9 software) following a methodology developed by Roelfsema *et al*.^89^. Satellite data, including sub-surface and seafloor reflectance as well as tide-corrected water depth to mean surface level were derived from a mosaic of 8 Landsat 8 Operational Land Imager scenes, pan sharpened from 30 m x 30 m to a 15 m by 15 m resolution, and combined with wave models (simulating waves nearshore (SWAN) model^74^), field data^90^ and satellite derived bathymetry to generate a geomorphic map layer, following a ruleset based on *Reef Cover*. A full methodology is available in Roelfsema *et al*.^36^.

A restricted geographic range and local knowledge about geomorphology enabled the researchers to apply hard thresholds to each class which allowed data to be assigned to a particular *Reef Cover* class. For example, a significant wave height of >2 m was set as the threshold for a *Reef Slope* (“Fore reef exposed”) and <2 m as a *Back Reef* class, while a 0.75 m depth threshold was used to distinguish *Reef Crest*, *Outer Reef Flat* and *Inner Reef Flat* from other classes^30^. Slope angle thresholds (e.g. 5–90 degrees for a *Reef Slope* or *Sheltered Reef Slope*) and sub-surface reflectance thresholds were also set, and these rules were then used to assign data to geomorphic classes.

#### Case Study #2. Remote reef mapping in Micronesia for conservation

**Fig. 6:**
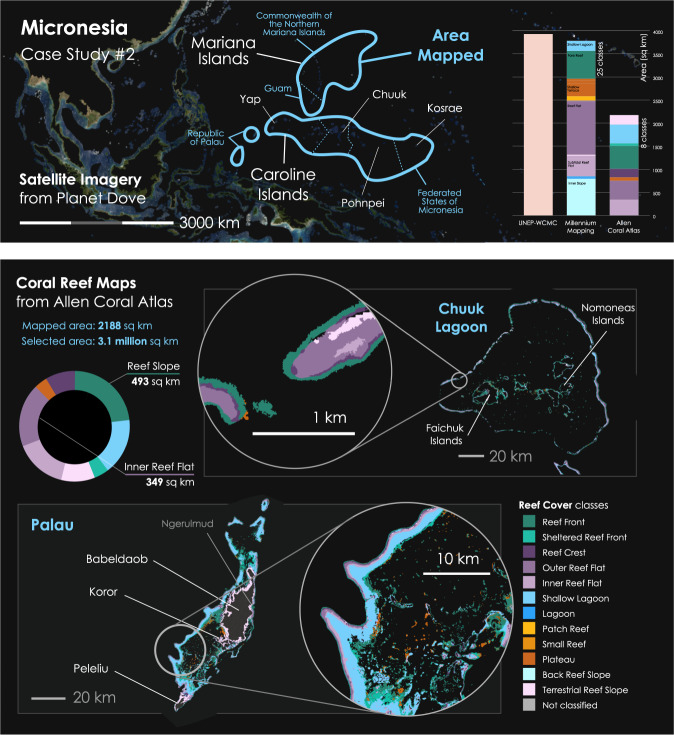
In Case Study 2, the *Reef Cover* geomorphic classification scheme was applied to an international coral reef mapping exercise: taking 3,072,192 km^2^ of spectral reflectance data (top panel) to generate coral reef habitat maps for reefs across Micronesia (e.g., Palau and Chuuk Lagoon, bottom panel). Over 2000 km^2^ of shallow coral reef of the Caroline Island chain (Republic of Palau, Federated State of Micronesia) and Mariana Island archipelago (Commonwealth of the Northern Marianas and US Territory of Guam) was mapped. Source data limited the number of classes that could be used (12/17 classes used) but in general corresponded well with the 25 classes mapped by *Millennium Coral Reef Mapping Project* (barchart).

The second case study is an NGO-led large scale mapping exercise to convert 20TB of daily remote sensing data captured by Planet Dove satellites from across a three million km^2^ area, into a sharable online habitat map of some of the planets remotest coral reefs^19^ (Fig. 6).

Two remote island chains in Micronesia were targeted. The Mariana Islands are a crescent-shaped archipelago of 15 volcanic islands making up the Commonwealth of the Northern Marianas (CNMI) and the Territory of Guam. The Caroline Islands, an archipelago of over 500 small islands, span 3500 km from Palau’s Hatohobei Reef in the westernmost point to Kosrae (Federated States of Micronesia) in the east. Spread across a 3 million km^2^ area, the wide dispersal of these isolated reef systems makes them challenging to map using traditional in-water surveys, and regional mapping to date has relied on earth observation data. Large parts of Micronesia have been mapped by collation of existing maps (*ReefBase*’s Pacific Maps) and through detailed bathymetry surveys of US jurisdictions of the Mariana Islands by NOAA’s *Pacific Islands Benthic Habitat Mapping Center* (PIBHMC). The *Millennium Coral Reef Mapping Project* (MCRMP) used Landsat data to map 6000 km^2^ of reef across Palau, Federated States of Micronesia, the Marshall Islands and Gilbert Islands^91^ and the Japanese Ministry of the Environment mapped the region using AVNIR2 and Landsat data in 2010^78^. Many of these maps were used to produce the United Nations Environment Program (UNEP) World Conservation Monitoring Centre (UNEP-WCMC) *Global Distribution of Coral Reefs* data product, that is used as a global coral extent layer today^86^.

The *Reef Cover* classification was used to generate coral reef habitat maps of the Marianas and Caroline Islands from satellite data, using an automated cloud-based machine learning approach and combined with object-based clean-up developed by Lyons, *et al*.^38^. Sentinel-2, Landsat 8 and Planet Dove satellite-derived relative bathymetry and surrogate for slope angle were used to identify major structural classes such as lagoons or reef slopes. In contrast to Case Study 1, hard thresholds were not applied to *Reef Cover* but training data for the classifier and neighbourhood rules (e.g., reef crest cannot be surrounded by reef flat) were more important in determining class. Expert derived, manually interpreted training data were used to train an algorithm to identify *Reef Cover* classes from the data. Combined with information on the substrate, calculated from colour, brightness levels and texture produced by spectral reflectance data from the satellites, geomorphic classes were assigned (Fig. 6). All code for developing these maps is publicly available^87^.

### Assessment: challenges and benefits of *Reef Cover*

In both case studies, *Reef Cover* was adapted to suit the specifics of the mapping exercise. Not all *Reef Cover* classes were used^23^: in Case Study #1, spatial resolution of the satellite data meant *Wall* and *Sheltered Wall* classes were not detectable. In Case Study #2, bathymetry was only reliable to a depth of 10 m, so the deeper *Reef Slope* and *Sheltered Reef Slope* classes were additionally beyond detection limits, and *Reef Island* was not included due to the way the land masking was performed (Table 3). Turbid water in inshore areas led to the requirement for a *Terrestrial Reef Flat* class (like fringing reef flat, but directly attached to land and subject to freshwater runoff, nutrients and sediment).

#### Case Study #1

A total of 1,984 km^2^ of shallow water coral reefs were mapped from Innisfail to Lizard Island, with 15 of the 17 *Reef Cover* classes used (*Wall* and *Sheltered Wall* not used). This allowed for a refinement of the total reef area estimate for this region from 3,164 km^2^ to 1,984 km^2^ (Fig. 5). Dominating in terms of areal extent was *Back Reef Slope* (23% of habitat), *Outer Reef Flat* (15%)*, Inner Reef Flat* (15%) and *Reef Crest* (12%) which together made up >60% of the shallow mapped reef area^36^, reflecting the broad flat-topped nature of the platform reefs typical of the Queensland shelf. Following the release of these maps to GBRMPA (the governing body), several applications that could directly support management have been identified, from improved understanding of coral ecological and biophysical processes through much refined extent layers to helping select appropriate areas for reef restoration and adaptation (see Roelfsema *et al*. 2020 for details)^36^.

#### Case Study #2

A total of 2,188 km^2^ of reef were mapped, with eight classes used (Fig. 6, Table 3). Dominating in terms of areal extent were *Reef Fron*t (renamed Reef Slope) comprising 23% of the shallow mapped reef area, the *Inner* and *Outer Reef Flat* classes (together 35%) and *Shallow Lagoon* (18%), reflecting that the region is typified by atolls or high islands with or without expansive lagoons. The spread of classes was largely in agreement with MCRMP outputs, who mapped more than twice as many classes (24 classes at the L4 level which aligns best with *Reef Cover*), but found five of these (Bay Exposed Fringing, Inner Slope, Fore Reef, Shallow Terrace and Subtidal Reef Flat) made up 85% of the map extent suggesting at very large scales some of the additional detail is redundant. A crosswalk found the distribution of classes was comparable with the *Allen Coral Atlas* classes, with *Reef Front* (MCRMP *Forereef*) accounting for 16%, *Inner and Outer Reef Flat* (seven classes combined) 43% and *Shallow Lagoon* (three classes combined) 30%. The total reef area mapped by MCRMP across the same area was 15,808 km^2^ and 38 classes at the L4 level (see crosswalk Online-Only Table 2), but once the dataset was filtered to remove deep classes (*Deep Reef*, *Deep Non-Reef*) and *Land*, this became 3,809 km^2^ and 24 classes, the higher extent value reflecting MCRMP’s ability to map deeper (e.g. 37 km^2^ of *Deep Lagoo*n was mapped, while no *Deep Lagoon* was detected by the Allen Coral Atlas) and the inclusion of “Variable Depth” classes in this number.

Shallow reef extent estimates for the whole region were closer in value: *Allen Coral Atlas* estimated 1,569 km^2^ of *Reef Top*, MCRMP 1,293 km^2^ of *Shallow Reef* and UNEP-WCMC, who provide an outline of shallow reef, estimated an areal coverage of 1,590 km^2^ of shallow reef area.

A team of eight experts involved in the creation of the maps were asked to evaluate their confidence in assigning *Reef Cover* classes (Table 3). Scoring confidence in each class (from 1 *very low confidence* to 10 *very confident*) allowed assessment of how applicable map producers working with satellite data found the *Reef Cover* classes. While all classes were scored > 5, there was some variability in confidence between classes, with mappers having the greatest confidence and consistency in their ability to determine *Reef Crest* (mean score 8.0 ± 1.3 SD) and *Lagoon* (8.0 ± 0.9 SD) from remotely sensed data, and the least confidence around *Back Reef Slope* (5.9 ± 1.7 SD).

Application of the *Reef Cover* classification allowed us to test the usability of the typology to map producers, and it was generally found to be flexible and adaptable, and mappers more confident than not in interpreting and applying classes. It could be aligned with existing schemes, but with just 17 options remained simple enough to allow for automated processing at 1000 km + scales with minimal human supervision. While limitations of the source data meant not all *Reef Cover* classes could be captured in the exercises, retaining classes like *Wall* and *Slope* in the classification is important for producers who may be work with source data with finer spatial resolution or depth. Moreover, the classification proved a good foundation for development of both sets of maps, despite the large differences in reefs being mapped (shallow coastal platform reefs vs remote oceanic atolls), the approaches used (object-based image analysis in mapping software vs automated cloud-based processing in Google Earth Engine) and the rationale (national management vs international conservation), and maps created are pushing the boundaries of in terms of detail, consistency and spatial extent.

### Outcomes: use of maps developed using *Reef Cover* to address real-world challenges

The overarching purpose of developing the *Reef Cover* classification was to improve the conversion of large amounts of remote sensing data into a mapped format that not only could visually communicate reef information, *but would practically support coral reef science and conservation work*. The final test of *Reef Cover* is whether the classification aided translation of remote sensing data into a real-world practical outcome for users, with success measured in scientific and conservation outcomes.

#### Case Study #1

The Great Barrier Reef (GBR) is of immense biological, cultural and economic importance^92^, but has also rapidly degraded over the last 30 years, and managers are under pressure to halt the declines that have been attributed to global warming, cyclones, pollution and Crown-of-Thorns Starfish outbreaks^93^. Improved spatial information on reef geomorphic zonation is critical to support scientific work to understand how the GBR is changing, and to support resource management decisions that enable conservation of the reef and its essential ecosystem services. *Reef Cover* here supported production of geomorphic maps specifically to support governance for a significant portion of GBR shallow reefs at a level of detail not previously available. Compared to existing maps that only outline each reef, the increase in detail provided by these new habitat maps enabled discrete characterisation of each reef’s geomorphology and benthic composition. With the new habitat maps, areas within each reef can be identified as either coral habitat or not coral habitat.

Creation of these Cairns Management Region maps has led to refinement of coral and Crown-of-Thorns recruitment modelling and water quality modelling exercises, both of which were previously using reef extent maps, many of which included large areas of deep water overestimating reef size and distribution^36^. Living coral cover remains a key metric for managers assessing reef health, and in this Case Study the *Reef Crest*, *Reef Slope* and *Reef Front* classes were further used as a basis to derive an estimate of habitat available for coral colonisation (‘potential coral habitat’) – a value of 761 km^2,36^.

#### Case Study #2

The area mapped in Case Study #2 is home to 353,000 islanders whose culture, and many cases livelihoods, are related to coral reefs though fishing, recreation and tourism. These reefs continue to be impacted by pressures from climate change (warming and high intensity storms) and local pollution (associated with crown-of-thorns sea star outbreaks) and overfishing^80^. The six nations remain committed to the *Micronesia Challenge*, an international conservation strategy across the six nations of Micronesia to conserve 30% of marine resources by 2020 in line with UN Sustainable Development goals. Mapping can help support marine spatial planning exercises: habitat maps such as the MCRMP of the area have been previously used to calculate estimates of fish biomass^81^.

In the production of the Micronesia maps, 125 GB of remote sensing spatial data (extracted from over 20TB of raw Planet Imagery) was converted into eight *Reef Cover* classes and displayed in an interactive web platform at AllenCoralAtlas.org. These products are now being used to a) communicate coral reef information and b) support conservation planning by coral reef practitioners in the region, from NGOs and research organisations to the communities that live on the islands. For example, maps of reefs in the Republic of Palau have been integrated into a spatial analysis model by *The Nature Conservancy*, to help the organisation identify where to site aquaculture as part of a national-scale project in partnership with the *Palau Environmental Quality Protection Board*, *Bureau of Marine Resources* and *Palau Community College*. Meanwhile, maps of the reefs across the Federated States of Micronesia are being used by the *Waitt Institute* to help plan research expeditions to remote submerged reefs, as well as support marine spatial planning exercises in the area. Researchers commented that, “*for a place like the FSM that has a pretty crude current estimate for distribution of coral reef structures, the Atlas is a huge value for them and a very necessary tool in the marine spatial planning process*” and “*These are some of the most critical pieces of information to create policy*.” In Micronesia’s Outer Islands, *One People One Reef*, a collaboration of communities and scientists, believed the open-access and easy to use format of the products could prove useful in communicating ideas and connecting with remote communities involved in traditional management. Finally, researchers leading the *Micronesia Challenge* at the University of Guam found the maps to be a dynamic and interactive communication tool during stakeholder meetings, allowing meeting participants to design and review current marine protected areas, and negating the need for an on-site GIS expert during meetings.

There is still room to improve both the mapping process and user experience: the number of classes used in the typology was refined due to data limitations. A survey of 26 users found 42% of those frequently worked in places with little or no internet connectivity, and 10% needed to work on a mobile device^82^. On balance, use of *Reef Cover* helped creators generate large scale consistent maps from remote sensing data, and map classes proved relevant and useful for researchers and managers looking to solve management and conservation challenges.

## Usage Notes

### Using *Reef Cover* to create coral reef maps

Users planning to create their own coral reef geomorphic maps using the *Reef Cover* classification system (Version 1.0) presented should download and read though the full “*Reef Cover Classification (v1). Internal coral reef class descriptors for global coral reef habitat mapping*” available as a .pdf through Dryad^23^.

**Fig. 7:**
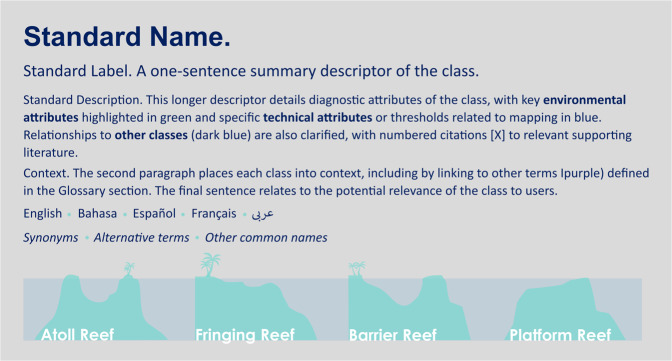
How to use *Reef Cover*: the pdf document walks through each of the 17 *Reef Cover* classes providing information on five variables: Standard Name - short class name, Standard Label - longer class name, Standard Description – detailed class descriptor, including context and main attributes (highlighted), Translations - Standard Name in different languages, Synonyms - list of commonly used synonyms.

The document provides detailed information on how to define and interpret each of the 17 suggested Geomorphic Reef Classes (Fig. 7), with additional information on how each of these classes can be related to physical attributes (Table 1), colour and texture (Fig. 3) and relational characteristics (Table 2), while the in-depth descriptors allow flexibility in interpreting classes for different user needs. Depending on the quality and type of your input data, users might want to consider setting and applying thresholds for attributes such as slope angle and depth to facilitate mapping (as in Case Study 1), or excluding non-relevant classes such as those are beyond mapping detection limits (as in Case Study 2). An example decision tree of how users might determine geomorphic map classes is also provided below (Fig. 4).

Diagrams of how each class relates to major reef types (atolls, fringing reefs, platform reefs and barrier reefs e.g. Figure 7), and a Glossary of terms is also provided.

Finally, for additional support in creating maps using *Reef Cover*, the Google Earth Engine code used to produce the Micronesia maps presented in this Case Study 2 is fully open access (https://github.com/CoralMapping/gee-mapping-source) and in full detail at 10.5281/zenodo.3833246^87^.

### Using *Reef Cover* to interpret coral reef maps

As well as using the *Reef Cover* classification document as a basis for creation of new maps from remote sensing, the classification is also intended as a guide or key for map users in the conservation space wanting to interpret the Great Barrier Reef^24^ or Micronesia maps^88^ or any future mapping products (e.g., Allen Coral Atlas habitat maps^17^) that are created around the classification.

Users planning to interpret geomorphic mapping products for their own scientific or conservation goals should also download and read though the full “*Reef Cover Classification (v1). Internal coral reef class descriptors for global coral reef habitat mapping*” available as a .pdf through Dryad^69^, to get a full understanding of how classes can be interpreted.

To further support users, a free online course, *Remote Sensing and Mapping for Coral Reef Conservation Online Course*, designed to help marine managers, conservation practitioners, scientists, decision makers, and GIS professionals decide whether these remote sensing products and mapping technologies can help inform their conservation and restoration work. Lesson two is developed around *Reef Cover* and provides more information in how to interpret the *Reef Cover* map classes.

#### Using Case Study 1 (Cairns Management Area) maps

Static geomorphic digital maps of the Cairns to Cooktown management area of the Great Barrier Reef are downloadable as a shapefile from 10.1594/PANGAEA.925657. This dataset consists of shapefiles and auxiliary files for each of the geomorphic zonation and dominant benthic cover types on the shallow offshore reefs of the Cairns to Cooktown Management area of the Great Barrier Reef, Australia. See Table 3 for how *Reef Cover* class definitions link to Cairns Management Region map class names.

#### Using Case Study 2 (western Micronesia maps), and other Allen Coral Atlas resources

Static geomorphic digital maps of shallow coral reef habitat across the Caroline and Mariana Islands in western Micronesia (Version 1.0) are downloadable in several formats as a tar file (western-micronesia-aca.tar.gz) from Zenodo^19^ [10.5281/zenodo.3953052]. MacOS can open tar and tar.gz files by default with the Archive UtilityUsers, Windows users will need an external program (7-Zip or WinRar) to extract files. The compressed folder *Western-Micronesia* contains three sub-folders: *geomorphic*, *benthic* and *boundary*. The *geomorphic* folder contains digital geomorphic coral reef habitat maps described in this study, mapped to 12 Allen Coral Atlas *Reef Cover* classes (Fig. 5, class descriptor available in *Reef Cover* document^69^) and stored in a variety of standard geospatial formats. Shapefiles are available for use with most GIS software (geomorphic.shp, geomorphic.shx, geomorphic.dbf and geomorphic.prj), a KML file for viewing in a browser such as Google Earth (geomorphic.kml), and GeoJSON (geomorphic.geojson). The *benthic* folder contains the same file formats, but same reefs have been mapped to six benthic classes: *Seagrass*, *Coral Habitat*, *Rubble*, *Sand*, *Microalgal Mats* and *Rock* (see *Reef Cover* for class descriptors^69^), and the *boundary* folder contains the outline of the area mapped, again in the same three formats. Users will be able to access updates and download further regional maps through via this Zenodo link [https://zenodo.org/record/3833246#.Xxo1togzZPY] which links through dynamic maps currently hosted to AllenCoralAtlas.org [http://www.allencoralatlas.org/]^18^, where up-to-date usage notes are available through the “FAQ” section. Further support is available by emailing support@allencoralatlas.org. The Allen Coral Atlas currently has two mapped themes: one that displays *global geomorphic zones* (12 classes) and another for *global benthic zones* (6 classes) commonly associated with shallow water tropical coral reefs. See Table 3 for how *Reef Cover* class definitions link to Allen Coral Atlas class names.

Full methodology for creating your own maps following the Great Barrier Reef mapping methodology are available here. The *Allen Coral Atlas* classification process and links to the code used to produce the maps, along with the data themselves can be accessed through *Zenodo* link [10.5281/zenodo.3833246]^87^. This link provides resource and citation for code, standards and publications arising from the *Allen Coral Atlas*^18^, including Google Earth Engine source code for mapping algorithms: https://github.com/CoralMapping/gee-mapping-source. This repository contains all the Google Earth Engine source code that generates the mapping outputs on the *Allen Coral Atlas*, including maps and validation statistics.

## Data Availability

The Google Earth Engine source code used to produce the Micronesia maps presented in this dataset (and all regions) is fully open access (https://github.com/CoralMapping/gee-mapping-source) and in full detail at Zenodo^87^ [10.5281/zenodo.3833246].

## Online-Only Table

**Online-Only Table 1:** Summary of some of the major contemporary coral reef classifications designed for global mapping, monitoring and management applications across very large scales (regional-global).

System	Primary application^1^	Source data	Scope and coverage	Type	Levels	M	G	E	Units	Description
Indo-Pacific Coral Reef Classification *Battistini et al. 1975*^7^	D	Morphological (aerial photos)	Indian Ocean and Pacific Ocean	Hierarchical	2	23	99	—	125	Battistini and 24 experts used morphology to define 125 broad reef types in a 2-level typology: L1 included 23 reef types (fringing reef, boat channel, barrier reef, double barrier reef, sand cay reef, coral bank, coral head, atoll, almost atoll, faros, emerged reef, drowned reef, lagoon, coral reef complex, while L2 defined subfeatures across three zones, fore-reef (14 classes), reef flat (66 classes) and lagoon (19 classes), translating into English, French and German. Recently adapted, merging with Millennium Classification to create detailed global classification^85^
Marine Ecosystem Classification System (MECS) *Holthus & Maragos 1995*^8^	A	Ecological	Regional South Pacific*	Hierarchical, nested	6	3	2	1	>300	A detailed classification system describing A) pelagic and B) benthic marine features. Within the “Benthic” typology there are six levels (L1-L6), with “ecological units” being the lowest level. L1 splits between Oceanic Reefs and Continental Shelf Reefs, and further by Reef Type (e.g. fringing, atoll) before assigning a geomorphic classes and finally ecological (e.g. seagrass, mangrove etc).
Geomorphological Classification of Reefs (NESP) *Nichol et al. 2016*^84^	A	Bathymetric (LiDAR)	Regional Australia	Non-hierarchical	7	—	7	1	>100k	Australian Government’s *National Environmental Science Programme* developed for managing continental shelf reefs (biogenic and non-biogenic). The classification draws heavily on the US Coastal and Marine Ecological Classification Standard (CMECS)^94^. 7 categories include L1 Reef Origin (4 classes), L2 Climatic Region (5), L3 Shelf Zone (4), L4 Geofeature (12), L5 Relief (4), L5.1 Slope (6), L6 Rugosity (6) and L7 Substrate (4), with further sub-divisions below substrate. Because the system is non-heirachical any number or combination of categories can be used to describe a reef.
Morphology of Recent Coral Reefs *Stoddart 1969*^17^	D	Review	Global		1		10	—	10	Stoddart reviewed existing reef zonation studies and suggested a global reef zone classification largely derived from zonation schemes for windward reefs by Ladd *et al*. 1950, and Tracey, Cloud & Emery 1955. With one level and 10 zones (*Seaward Slope*, *Terrace*, *Reef Front*, *Algal Ridge*, *Boulder Zone*, *Reef Flat*, *Atoll Land*, *Narrow Lagoon Flat*, *Lagoon Reef Slope* and *Lagoon Floor with Reef Knolls*) Stoddart is also careful to describe regional differences.
Geomorphic Zonation *Blanchon 2011*^71^	D	Review	Global		2		5	—	5	Blanchon’s entry for the *Encyclopaedia of Modern Reefs* describes five “standard first order” geomorphological zones (L1: *lagoon*, *flat*, *slope*, *front* and *apron*) divided by demarcation breaks in slope angle, and several “second order” subzones (L2: e.g., Coralgal Flat), which are described in detail.
Reef Cover Classification System (GBRMPA) *Kuchler 1986*^57^	A, B	Review	Regional Australia	Non-hierarchical	5	—	2	3	>100k	Commissioned by Australia’s *Great Barrier Reef Marine Park Authority* (GBRMPA) to standardize classification of the Great Barrier Reef for the purpose of mapping. The classification reviewed decades of commonly used geomorphological nomenclature before designing a 5-level typology: L1 Zones (36 classes), L2 Features (41), L3 Composition (39), L4 Condition/Morphology (4 levels) and L5 Presence (depths and percent covers, 4 levels). The classification is not strictly hierarchical so a mapping unit could fall into multiple categories.
Millennium Coral Reef Mapping Project classification *Andréfouët et al. 2006*^21^	B	Morphological (satellite data)	Global (80% coverage, >200 maps)	Hierarchical, nested	5	3	2	—	800	MCRMP defined a globally standardised coral reef geomorphological typology based on satellite (Landsat 7) imagery. A five-level classification system, L1 (Main Division, 2 classes), L2 Nodes (12), L3 Blocks (68), L4 Geomorphological (126) to L5, the most detailed description scheme, a list of 800 different codes and habitats, which are defined through unique combinations of L1 to L4 features.
World Atlas of Coral Reefs *Spalding, Ravilious & Green, 2001*^28^	B	Published maps	Global (249,713 km^2^)		1	6	5	—	—	The *United Nations Environment Program World Conservation Monitoring Centre* (UNEP-WCMC) began its global coral reef mapping work in 1994, eventually publishing the *World Atlas of Coral Reefs*: Because of the different data sources a map a uniform classification was not possible, but the *World Atlas of Coral Reefs* suggests key reef classes in the accompanying book and dataset.
Living Oceans Foundation *Purkis et al. 2019*^49^	B	Satellite and field data	Global 11 countries (65,029 km^2^), >1000 reefs	Hierarchical, aggregated	5	—	3	2	36	Developed for the LOF’s Global Reef Expedition for the purpose of supporting mapping of 65,000 sq km of remote reefs^49^, using combined satellite, bathymetric and ecological data. Five levels include benthic and geomorphic components: L1 Zones (8 classes, based purely on bathymetry), L2 Major Geomorphological Structure (3), L3 Detailed Geomorphological Structure Map (11) together form the hierarchical classification, then L4 Biological Cover (from field surveys) and L5 Aggregate Map Class (36 classes).
NOAA Biogeography Reef Mapping Program* *Monaco et al. 2012*^50^	B	Satellite and field data	Global US territories (43,000 km^2^)	Hierarchical, nested	5	—	2	3		The USA’s *National Oceanic and Atmospheric Administration* (NOAA) National Ocean Service developed a series of hierarchical geomorphic habitat classification schemes to support a reef habitat mapping effort, aimed at mapping 43,000 sq km of coral reef across the US territories. Classifications had some regional variations with five classifications developed for the seven mapped regions but largely followed the same format, with five levels: L1 Geographic Zones (~14 classes), L2 Geomorphological Structures (~12), L3 Biological Cover types (~7), L4 Coral Cover categories (4 classes: 10–50%, 50–90%, 90–100% or unknown) and L5 Percent Hardcover categories (4)^69^. This classification also feeds into the US Coastal and Marine Ecological Classification Standard (*CMECS*) published by the Federal Geographic Data Committee^94^, which describes 10 similar reef classes to level L2 (Back Reef, Bank/Shelf, Bank/Shelf Escarpment, Fore Reef, Lagoon, Reef Crest, Reef Flat, Vertical Wall, Shoreline/Intertidal and an extra class, Ridges and Swales).
Atlantic and Gulf Rapid Reef Assessment (AGRRA) *Lang & Marks 2018*^56^	C	Ecological data	Regional Caribbean	Hierarchical, aggregated	4	2	1	1		AGRRA is a Caribbean-specific classification developed to support field monitoring purposes and guide surveywork. The “Reef Type” classification is comprised of four-levels: L1 Location (6 classes), L2 Reef Type (15), L3 Geomorphic Zone (9), L4 Habitats (9) which can be combined with 14 Attribute Classes related to exposure, incline and relief) to describe the reef type being monitored.
Reef Check classification *Sully et al. 2019*^58^	C	Ecological data	Global Atlantic/Caribbean, Arabian/Persian Gulf, Hawai’I, Indo Pacific and Red Sea		1			1	10	Reef Check, a global citizen science monitoring program, trains surveyors to classify benthos into 10 substrate categories (e.g., *Hard Coral*, *Soft Coral*, *Recently Killed Coral*, *Nutrient Indicator Algae*, *Sponge*, *Rock*, *Rubble*, *Sand*, *Silt/Clay* and *Other*) for the purpose of monitoring change on coral reefs. Modified slightly for the five regions: Atlantic/Caribbean, Arabian/Persian Gulf, Hawai’I, Indo Pacific and Red Sea.
Global Coral Reef Monitoring Network (GCRMN, Caribbean) *Wilkinson 2008*^95^	C	Ecological data	Global		2			2	106	GCRMN is a global network of reef researchers working to improve understanding of coral reef status and trends, globally and regionally, by coordinating collection and aggregation of monitoring data. Caribbean methods which draw from AGRRA and Reef Check^96^ vary regionally e.g. Caribbean method has two levels (L1: Coarse benthic, including *Stony coral*, *Macroalgae*, *Turf Algae*, *Cyanobacteria*, *Gorgonians*, *Rubble*, *Sand*, *Limestone free from growth* and *Other*, and L2 Detailed categories (106 categories including coral species).
Coral Triangle Initiative, *Coral Triangle Initiative on Coral Reefs 2013*^97^	A/C	Ecological data	Regional Coral Triangle (Indonesia, Philippines, Solomon Islands, Timor-Leste, Malaysia, Papua New Guinea)		4	1	3	—	180	Indonesia’s 1998 Coral Reef Rehabilitation and Management Program (COREMAP) was expanded to six Coral Triangle nations, with a monitoring protocol^59^ that focuses on four reef attributes (Reef Type (5 types, Atoll, Fringing, Lagoon, Barrier, Patch), Reef Slope (3), Reef Zone (3) and Exposure (4), and a map that highlights reef extent.

M = Morphological (“Reef Type”), G = internal geomorphological zonation (“Reef Zone”), E = Ecological units;

^1^A = Management, B = Mapping, C = Monitoring, D = Review of Scientific Understanding.

**but successfully adapted to other regions (e.g. Caribbean*^62^)

**Online-Only Table 2:** Crosswalk table comparing how some of the major regional to global reef mapping and monitoring efforts (detailed in Online-Only Table 1) classify internal reef structures and how categories relate to Reef Cover classes.

	Marine Ecosystem Classification System (MECS) [48] *classification*	Geomorphological Classification of Reefs (NESP) [59] *classification*	Reef Cover Classification System (GBRMPA) [36] *classification*	Millennium Coral Reef Mapping Project [46] *classification and regional maps*	World Atlas of Coral Reefs [10] *map classes*	Living Oceans Foundation [45] *map classes*	NOAA Biogeography Reef Mapping Program* [60] *map classes*	Allen Coral Atlas [8] *map classes*	Atlantic and Gulf Rapid Reef Assessment [45] *monitoring classes*
**Reef Slope**	✓ REEF SLOPE (NON-TERRACE) (L5): **REEF EDGE SCARP** (L6). *Orientation: windward + *+	✓ **SCARP** (L4), steeply sloping (L6)	✓ **REEF SLOPE** (L1) **(lower reef slope), windward** (L3)	✓ **SHELF SLOPE** (L4) + multiple L5 categories	✓ **REEF SLOPE/REEF FRONT**	✓ **BANK/SHELF ESCARPMENT** (L1) | **DEEP FORE REEF SLOPE** (L5)	✓ **BANK/SHELF ESCARPMENT** (L2)	×	✓ **OUTER FORE REEF** Attribute: *windward* or *seasonally variable*, *moderate* or *steep* slope
**Sheltered Slope**	✓ REEF SLOPE (NON-TERRACE) (L5): **REEF EDGE SCARP** (L6). *Orientation: leeward + *+		✓ **REEF SLOPE** (L1) **(lower reef slope), leeward** (L3)			×		×	✓ **OUTER FORE REEF** Attribute: *leeward*, *protected leeward* or *exposed leeward*, *moderate* or *steep slope*
**Reef Front**	✓ REEF SLOPE (NON-TERRACE) (L5) **SLOPE, SPUR AND GROOVE** *Orientation: windward + *+	✓ **LEDGE** (L4) **steeply sloping (L6)**	✓ **REEF SLOPE** (L1) **(upper reef slope) windward** (L3)	✓ **FOREREEF** (L4)	✓ **REEF SLOPE/REEF FRONT**	✓ **FORE REEF** (L1) | **SHALLOW FORE REEF SLOPE** (L5)	✓ **FORE REEF** (L2)	✓ **REEF SLOPE**	✓ **INNER FORE REEF** Attribute: *windward* or *seasonally variable*, *moderate* or *steep* slope
**Sheltered Front**	✓ REEF SLOPE (NON-TERRACE) (L5) **SLOPE, SPUR AND GROOVE** *Orientation: leeward + *+		✓ **REEF SLOPE** (L1) **(upper reef slope) leeward** (L3)					✓ **SHELTERED SLOPE**	✓ **INNER FORE REEF** Attribute: *leeward*, *protected leeward* or *exposed leeward*, *moderate* or *steep slope*
**Terrace**	✓ REEF SLOPE OUTER (L4) |, **TERRACE (L5) PLATFORM** – **SUBMARINE** (L6) + + 7	✓ **TERRACE** or **LEDGE** (L4)	✓ **TERRACES**	✓ **SHALLOW TERRACE** (L4)		✓ ✓ **FORE REEF** (L1) | **SHALLOW FORE REEF SLOPE** or **SAND FLATS** (L5)	✓ **BANK/SHELF** (L2)	×	✓ **OUTER** or **INNER FORE REEF** Attribute: *flat*
**Wall**	✓ REEF SLOPE OUTER (L4) | REEF SLOPE NON-TERRACE (L5) | **SUBMARINE CLIFF, SUBMARINE WALL** (L6) *Orientation: windward* + +	✓ **SCARP** (L4), vertical (L6)	×			×	✓ **VERTICAL WALL** (L2)	×	✓ **OUTER** or **INNER FORE REEF** Attribute: *wall*
**Sheltered Wall**	✓ REEF SLOPE OUTER (L4) | REEF SLOPE NON-TERRACE (L5) | **SUBMARINE CLIFF, SUBMARINE WALL** (L6) *Orientation: leeward*++		×			×		×	✓ **OUTER** or **INNER FORE REEF** Attribute: *wall*, *leeward, protected leeward* or *exposed leeward*
**Reef Crest**	✓ REEF TOP (L4) | **REEF TOP SURFACE FEATURES** (L5) + +	✓ **RIDGE** (L4)	✓ **REEF RIM** (L1)	✓ **RIM** (L3) / **CREST** (L4)	✓ **REEF CREST**	✓ **REEF CREST** (L1)	✓ **REEF CREST** (L2)	✓ **REEF CREST**	✓ **BREAKER ZONE**
**Outer Reef Flat**	✓ REEF TOP (L4) | REEF FLAT SURFACE FEATURES (L5) | **REEF PAVEMENT, CORAL BED, COBBLE FIELD + **+	✓ **PLATFORM** (L4) rock (L8)	✓ **OUTER REEF FLAT** (L1)	✓ **REEF FLAT** (L4)	✓ **REEF FLAT**	✓ **BACK REEF** (L1) | **CORAL REEF AND HARDBOTTOM** (L2) + +	✓ **REEF FLAT** (L2)	✓ **OUTER REEF FLAT**	✓ **FLAT ZONE**
**Inner Reef Flat**	✓ REEF TOP (L4) | REEF FLAT SURFACE FEATURES (L5) | **SAND, RUBBLE, ROCK FLATS (L6) + **+	✓ **PLATFORM** (L4) unconsolidated hard (L8)	✓ **INNER REEF FLAT** (L1)	✓ **SUBTIDAL REEF FLAT** (L4)		✓ **BACK REEF** (L1) | **UNCONSOLIDATED SEDIMENT** (L2) + +		✓ **INNER REEF FLAT**	✓ **BACK ZONE**
**Back Reef Slope**	✓ **REEF SLOPE - LAGOON** (L4) + 7 subclasses	✓ **LEDGE** (L4) **gently sloping** (L6) **unconsolidated soft** (L8)	✓ **BACK REEF ZONE** (L1)	✓ **BACK REEF** (L4)	✓ **BACK REEF**	✓ **SEDIMENT APRON** (2)	✓ **BACK REEF** (L2)	✓ **BACK REEF SLOPE**	✓ **SUBTIDAL CREST**
**Lagoon**	✓ **LAGOON FLOOR** (L4) + + 4 subclasses	✓ **DEPRESSION** (L4) **unconsolidated soft** (L8)	✓ **LAGOON** (L1)	✓ **LAGOON** (L3)	✓ **LAGOON**	✓ **LAGOON** (L1) | **UNCONSOLIDATED SEDIMENT** ( + + 4)	✓ **LAGOON**	✓ **DEEP LAGOON**	✓ **LAGOON**
**Shallow Lagoon**	✓ REEF TOP (L4) | REEF TOP SUBTIDAL FEATURES (L5) | **MOAT AND DEPRESSION + **+	✓ **CHANNEL** (L4)	✓ **SHALLOW LAGOON** (L1)	✓ **SHALLOW LAGOON WITH CONSTRUCTIONS / SHALLOW LAGOONAL TERRACE** (L3)		✓ **LAGOON** (L1) | **LAGOON FLOOR** (2)	x but see **CHANNEL**	✓ **SHALLOW LAGOON**	✓ **CHANNEL**
**Plateau**	✓ **SHOAL** + Submerged Atoll, Table Reef, Bank ( > 20 m) + +	✓ **BANK** (L4)	✓ **REEFAL SHOAL** (L1)	✓ **DEEP TERRACE** (L4)		×	✓ **BANK/SHELF** (L2)	✓ **PLATEAU**	✓ **PLATFORM**
**Patch Reef**	✓ **LAGOON REEF** (L4) | + + 3 subclasses (Patch Reef, Patch Reef + Islet, Reticulate Reef) + +	✓ **MOUND** (or **RIDGE**) (L4)	✓ **PATCH REEF** / **CORAL HEAD** / **MICROATOLL** (L2)	✓ **INTRA-LAGOON PATCH** (L3)		✓ LAGOON (L1) | **PINNACLE REEFS** ( + + 3), **AGGREGATE REEFS** (+ + 2), **PATCH REEFS** ( + + 2) (L5)	✓ **INDIVIDUAL PATCH**, **AGGREGATED PATCH REEF** (L3)	✓ **PATCH REEF**	✓ **SINGLE PATCH**, **COALESCED PATCH**, **LINEAR PATCH**, **RETICULATE PATCH**
**Small Reef**	×	✓ **KNOB** (or **MOUND**) (L4)	×	✓ **PATCH** (L2)		×	x but see Aggregate Reef	✓ **SMALL REEF**	✓ **SHOAL**
**Reef Island**	✓ **REEF ISLETS** + + 20 additional classes	×	✓ **CAY** (L1)	✓ **LAND ON REEF** (L4)		✓ **LAND** and **SHORELINE INTERTIDAL** (L1) + + 10 classes	x included in LAND and SHORELINE INTERTIDAL	**x** included in **LAND**	

✓ = same category and class terminology, ✓ = same meaning but different term (synonym), ✓ = similar term but slightly different meaning/interpretation (homonym), x = class not considered / not mapped / absent, ++ further subcategories available in classification *same as/similar to **Coastal and Marine Ecological Classification Standard (CMECS)**

**Marine Ecosystem Benthic Classification (*****MECS*****)**. MECS, a detailed classification system describing A) pelagic and B) benthic marine features, was developed in 1995 to accompany a broader South Pacific Ecosystem Classification System (SPECS). This classification is applicable outside of the South Pacific and has been successfully modified to other areas (e.g. Caribbean [23]). Within the “Benthic” typology there are **six levels** (L1-L6), with “ecological units” being the lowest level. This classification is highly detailed, Reef Cover classes relate most closely to MECS L4 classes. A major difference from Reef Cover is classes are first split between **Oceanic** Reefs and **Continental Shelf Reefs**, and further by **Reef Type** (e.g. fringing, atoll) before being assigned a geomorphic class.

**Reef Cover Classification System**. Kuchler’s 1986 classification system was commissioned by Australia’s Great Barrier Reef Marine Park Authority (GBRMPA) to standardize geomorphic classification of the Great Barrier Reef for the purposes of mapping using remote sensing data. The classification reviewed decades of commonly used geomorphological nomenclature before designing a typology with **five levels: L1 Zones** (36 classes), L2 Features (41), L3 Composition (39), L4 Condition/Morphology (4 levels) and L5 Presence (depths and percent covers, 4 levels). The classification is not strictly hierarchical so a mapping unit could fall into multiple categories. Reef Cover aligns best with L1.

**NESP Geomorphological Classification of Reefs**. The Australian Government’s *National Environmental Science Programme* (NESP) geomorphology classification scheme developed for managing continental shelf reefs (biogenic and non-biogenic) using bathymetric (LiDAR) data. The classification draws heavily on the US **Coastal and Marine Ecological Classification Standard** (CMECS) published by the Federal Geographic Data Committee [61]. **Seven categories** include L1 Reef Origin (4 classes), L2 Climatic Region (5), L3 Shelf Zone (4), L4 Geofeature (12), L5 Relief (4), L6 Slope (6), L7 Rugosity (6) and L8 Substrate (4). Reef Cover most closely aligns with L4 to L6.

**Millennium Coral reef Mapping Project (*****MCRMP*****)**. MCRMP defined a globally standardised coral reef geomorphological typology based on satellite (Landsat 7) imagery. A **five-level** classification system, L1 (Main Division, 2 classes), L2 Nodes (12), L3 Blocks (68), L4 Geomorphological (126) to L5, the most detailed description scheme, a list of 800 different codes and habitats, which are defined through unique combinations of L1 to L4 features (similar to Living Oceans Foundation Approach). Reef Cover aligns most closely with L3/L4 classes, intermediate geomorphological description level that reflects the main structures of a reef complex. Like the MECS and CMECS, MCRMP contains a high level of detail and is clearly split first between **Oceanic** Reefs and **Continental Shelf** Reefs, and further by **Reef Type** (e.g. fringing, atoll, barrier), but unlike the other schemes is strictly morphological and does not include benthic cover classes.

**World Atlas of Coral Reefs**. The *United Nations Environment Program World Conservation Monitoring Centre* (UNEP-WCMC) began its global coral reef mapping work in 1994, eventually publishing the *World Atlas of Coral Reefs*: the most comprehensive global extent map of coral reefs currently in existence in 2000 [10]. The map was derived from multiple sources, primarily maps supplied by the MCRMP at L3 (80% of the map), but also including existing US Defence Force navigational charts, and digitised regional topographic and specialised scientific reef maps (some as old as 150 years), collated as part of the *Corals of the World* volumes. Because of the different data sources a map a uniform classification was not possible, but the *World Atlas of Coral Reefs* suggests key reef classes in the accompanying book and dataset.

**Living Oceans Foundation*****Global Reef Expedition***. A hierarchical geomorphic classification scheme was developed for the Global Reef Expedition for the purpose of supporting mapping of 65,000 sq km of the world’s remotest reefs from 11 countries [53], using combined satellite, bathymetric and ecological data. Hierarchical classification that accompanies the maps have **five levels** that include benthic as well as geomorphic components: L1 Zones (8 classes, based purely on bathymetry), L2 Major Geomorphological Structure (3), L3 Detailed Geomorphological Structure Map (11) together form the hierarchical classification, then L4 Biological Cover (from field surveys) and L5 Aggregate Map Class (36 classes). The 36 classes include 6 shoreline intertidal class, 4 land classes. Reef Cover classes align best with L1-L5.

**NOAA Biogeography Reef Mapping Program**. The US *National Oceanic and Atmospheric Administration* (NOAA)‘s National Ocean Service developed a series of hierarchical geomorphic habitat classification schemes to support a reef habitat mapping effort, aimed at mapping 43,000 sq km of coral reef across the US territories. Classifications had some regional variations with five classifications developed for the seven mapped regions [60], but largely followed the same format, with **five levels**: L1 Geographic Zones (~14 classes), L2 Geomorphological Structures (~12), L3 Biological Cover types (~7), L4 Coral Cover categories (4 classes: 10–50%, 50–90%, 90–100% or unknown) and L5 Percent Hardcover categories (4) [11]. Reef Cover most closely aligns with L1, but misses key classes SALT POND, SHORELINE INTERTIDAL, DREDGED, CHANNEL and REEF HOLE. This classification also feeds into the US **Coastal and Marine Ecological Classification Standard** ***(CMECS)*** published by the Federal Geographic Data Committee [61], which describes 10 similar reef classes to level L2 (Back Reef, Bank/Shelf, Bank/Shelf Escarpment, Fore Reef, Lagoon, Reef Crest, Reef Flat, Vertical Wall, Shoreline/Intertidal and an extra class, Ridges and Swales).

**Allen Coral Atlas**. The Allen Coral Atlas developed by Vulcan has 18 map classes largely derived from a classification developed for reef mapping based on hierarchical rulesets based on environmental attributes for object-based image classification, which has four levels L1 Reef Type, L2 Reef Type, L3 Geomorphic (12 classes), and L4 Benthic (6) [52]. Reef Cover is similar to L3 Global Geomorphic Classes but shallow map depth means Reef Slope classes are missing and additional class TERRESTRIAL REEF FLAT also exists.

**Atlantic and Gulf Rapid Reef Assessment (*****AGRRA*****)**. AGRRA is a Caribbean-specific classification developed to support field monitoring purposes and guide surveywork. The “Reef Type” classification is comprised of four-levels: L1 Location (6 classes), L2 Reef Type (15), L3 **Geomorphic Zone (9)**, L4 Habitats (9) which can be combined with 14 **Attribute Classes** related to exposure, incline and relief) to describe the reef type being monitored. Reef Cover aligns well with the L3 classes, but is missing “FLANK”.
