# Author Correction: Reef Cover, a coral reef classification for global habitat mapping from remote sensing

**DOI:** 10.1038/s41597-021-01011-9

**Published:** 2021-08-31

**Authors:** Emma V. Kennedy, Chris M. Roelfsema, Mitchell B. Lyons, Eva M. Kovacs, Rodney Borrego-Acevedo, Meredith Roe, Stuart R. Phinn, Kirk Larsen, Nicholas J. Murray, Doddy Yuwono, Jeremy Wolff, Paul Tudman

**Affiliations:** 1 Remote Sensing Research Centre, School of Earth and Environmental Sciences, University of Queensland, Brisbane, Australia; 2 Australian Institute of Marine Science, Townsville, Queensland Australia; 3 Vulcan Inc, Washington, 98104 USA; 4 College of Science and Engineering, James Cook University, Townsville, Queensland Australia

**Keywords:** Macroecology, Geomorphology, Marine biology, Environmental sciences, Coral reefs

Correction to: *Scientific Data* 10.1038/s41597-021-00958-z, published online 02 August 2021

The original version of this text incorrectly cited reference 69 in the Usage Notes section of the article in relation to the authors’ data record at the Dryad repository. This has now been corrected to reference 23 in both the PDF and HTML versions of the Data Descriptor.

